# Comparative modelling of drone interventions with traditional logistics modes for expedited and equitable deliveries

**DOI:** 10.1038/s44333-025-00050-8

**Published:** 2025-08-25

**Authors:** Andy Oakey, Matt Grote, Aliaksei Pilko, Jakub Krol, Alexander Blakesley, Tom Cherrett, Antonio Martinez-Sykora, Bani Anvari

**Affiliations:** 1 University of Southampton, Southampton, UK; 2 University College London, London, UK

**Keywords:** Operational research, Engineering, Geography, Geography, Science, technology and society, Social policy

## Abstract

Uncrewed Aerial Vehicles (UAVs/drones) for deliveries have become a key consideration for logistics planners, particularly in the healthcare industry. Despite reported theoretical cost and speed benefits, few systems have realised long-term financially sustainable operations. This paper explores UAVs in a multi-mode logistics system (vans, UAVs, bicycles) using medical sample delivery case studies in the United Kingdom, and a computational model that accounts for routing, landing restrictions, costs, and payload constraint practicalities. Results identified potential transit time reductions of up to 90% using UAVs, though costs increased significantly (+133%). Achieving these time savings required UAV access to all sites and residents tolerating up to 40 flight movements per hour. Other time savings were possible with partial or no UAV uptake; however, all expedited solutions increased costs, raising the question of the value of such time savings, and whether any benefits would materialise, given onward supply chain limitations and changeable weather.

## Introduction

The increased use of Uncrewed Aerial Vehicles (UAVs, or drones) for commercial purposes over recent years has seen their application expand worldwide across numerous sectors, including monitoring and inspection, emergency response, photography, and surveillance. This versatility has led to interest in their potential for integration into logistics networks, particularly for last-mile delivery, where UAVs could offer several advantages over more traditional transport methods, such as Light Goods Vehicles (LGVs; usually known as vans or light-trucks) or cycle couriers^1–5^.

One suggested advantage of UAVs is their potential to expedite delivery times and enhance accessibility to remote or challenging locations, e.g., where existing surface transport infrastructure is poor or where large bodies of water or mountainous areas have to be traversed. UAVs have also been seen as potentially offering reduced energy consumption and associated greenhouse gas emissions (e.g., carbon dioxide) over traditional land-based modes^6,7^. However, there are likely to be cost implications associated with the use of UAVs, and despite the potential benefits, examples of commercial UAV logistics services on a routine, large-scale basis remain limited, likely hindered by factors such as economic viability and the need to navigate complex regulatory landscapes^6,8–13^.

In response, several investigations have sought to explore the potential for UAVs to be incorporated into multi-modal fleets, typically suggesting substantial benefits from these arrangements^14–17^; however, many factors relating to operational practicalities, airspace and routing issues, and costs are often omitted. Many of these factors have been highlighted as critical for accurately modelling such systems^12,18,19^, often impacting whether UAVs are viewed as beneficial or not.

Using case studies involving the movement of patient diagnostic samples from General Practitioner (GP) clinics to pathology analysis laboratories in urban, peri-urban and rural settings, this paper attempts to: i) identify the best combinations of vehicles when optimising between reducing operating costs and maximum transit times across all of the sites served; ii) quantify the scope for UAVs to reduce the maximum transit time of a pathology delivery system in the different environments; and (iii) understand the impact of site suitability and time constraints on the potential cost and time savings available within a logistics system. The relationship between cost and transit time was investigated using a comprehensive heterogeneous (multi-mode) time-constrained route optimiser^20^, and consideration of numerous practicalities including costs, UAV routing, consignment characteristics, and operational restrictions. The case studies covered areas across Southampton, UK (76 clinics in an urban/peri-urban environment), the Isle of Wight (IOW), UK (22 clinics in a peri-urban/rural environment), and the Scottish Hebrides, UK (23 surgeries in a large, island-based remote environment).

The remainder of this paper contains a review of current literature in this subject area; the methodology, including the case study characteristics; results from each of the examples and scenarios; and a discussion of the findings.

## Literature Review

The use of UAVs in logistics is a rapidly emerging area in both research and industry practice^21^, though there are few cases of successful implementation beyond a trial basis throughout the world^22,23^. A key advantage often cited by UAV logistics case studies is the prospect of improved delivery speeds over existing delivery methods, such as vans and cycle freight^7^, though evidence is emerging to suggest that this may not always be the case, and that there are often substantial caveats or trade-offs made when introducing UAVs, many of which relate to costs^10^, operational or regulatory constraints^12,22^.

The general impact of improved logistics in healthcare on care quality and outcomes has been well documented, with optimisation, innovation, and effective management of healthcare logistics throughout the supply chain being highlighted as key drivers of improved patient outcomes and patient satisfaction^24–27^. To this end, healthcare logistics is a commonly supported UAV application which may exploit the reported benefits of expedited delivery across diagnostic specimens^28,29^, blood stocks^30^, vaccines/medicines^31–33^, and medical equipment and emergency defibrillators^7,34^. It has been suggested that the theoretical time savings that could be realised through a UAV-supported logistics system may expedite the analysis of diagnostic samples^35^, facilitate demand-centric, just-in-time delivery for cold chain items^36,37^, or improve rapid response in life-critical situations^7,34^. It has also been suggested that many reported benefits of introducing UAVs may have been conflated and presented in a misleading manner^38^, or that their introduction will require trade-offs in other areas such as cost^10^.

A notable example of a well-established medical delivery service is Zipline. The company initially launched operations in the African nation of Rwanda, before expanding into other countries and continents^39^. The initial service offered a delivery of blood stocks for transfusion, citing the opportunities to reduce wastage as a key driver. Under a model of centralised stock storage, rapid delivery of products was a critical factor to enable the service, although it is not clear whether a UAV was essential in all situations^40^. Furthermore, it is understood that the costs of running the system are substantial and these have been borne by the government using philanthropic donations, bringing into question its long-term viability^41^. Zipline have since expanded operations into other countries and have diversified their service, offering delivery of other products, such as restaurant food and groceries, although the implications of this are as yet unknown.

While proof-of-concept trials and growing support for medical applications demonstrate the potential of UAVs, there is concern that these use cases are being strategically employed to cultivate public acceptance before expanding operations into other applications^38^. Sensationalised media coverage and overstated claims of benefits contribute to a misleading public perception and a potentially damaging and unethical approach^42^ to NHS and other proof-of-concept trials^43,44^. There are also examples of poorly evidenced claims of benefits to healthcare use cases related to cost, emissions, and time savings, with ties to the healthcare use cases or reputable publications being exploited to enhance the credibility and prominence of the claims^43,45–49^. Similarly, the UK government has heavily cited privately produced reports on the benefits and future of UAVs^50–52^, including healthcare benefits.

Academic policy research has also found that private sector reporting and analyses of UAV trials and their introduction are being poorly scrutinised before being cited as supporting evidence for widespread policy decisions^43,53^. A notable example of this is the use of inflated transit time savings within trial planning applications^54^. The problem has been further exacerbated by some academic research^55,56^, developing an effect similar to an echo chamber commonly seen on social media^57^. As a result, it is difficult to understand the true extent of how beneficial UAVs would be in real-world situations. Furthermore, with the slow emergence of healthcare-based UAV trials, anecdotal commentary by industry practitioners has started to question the validity of UAV delivery use cases and has highlighted that UAVs may not be the solution in many situations, with care quality and equity challenges being caused by more than just delays to a small component of the supply chain^58^. Insights are also emerging around social equity concerns related to the use of UAVs in urban logistics^59^, including disparities around public safety, environmental impacts, inclusivity, accessibility, and affordability.

With respect to specific healthcare use cases, UAV trials by SwissPost concluded that: “Operations demonstrated that UAVs are well suited to urgent transportation of special items and that there is a clear interest in the service. However, UAVs are cost-intensive for Swiss Post and cannot be operated profitably in the medium term.”^60^, after having experimented with deliveries of a range of items, including several healthcare products. They also highlighted the potential benefits to healthcare given the urgent nature of some of these consignments, though they also noted the importance of considering UAVs as a supplemental service to other (more traditional) transport modes^60^, though they did not suggest how this would be best managed. Swoop Aero, a major UAV manufacturer and operator who specialised in healthcare deliveries to remote areas, has also faced financial challenges despite becoming well established^61^, aligning with the scaling challenges seen at SwissPost and other companies.

Similarly, Schreurs and Steuwer^19^ and Haidari et al^18^. used desktop studies and suggested that there would be substantial opportunities with respect to the delivery of vaccines and defibrillators, respectively, though there were numerous technological challenges to address, and costs would need to be offset by significantly higher delivery frequencies, potentially limiting the scope for adoption. Additionally, any additional benefits theoretically offered by UAVs in terms of greenhouse gas emissions reductions may be negated by the increased usage frequencies, worsening the current emissions footprint of global healthcare systems^62,63^. This could be likened to the Jevons Paradox (an increase in demand resulting from efficiency improvements negating any original saving); an issue that has already been noted for other UAV applications^64–66^.

To address these challenges, several studies have sought to identify how UAVs could operate as part of an integrated logistics system, with investigations such as Murray and Chu’s^14^ flying sidekick model proposing solutions (a combined van-UAV system where a UAV takes off from a van to complete deliveries whilst the van and driver continue, before later rendezvousing for further deliveries). Further developments of this arrangement have been explored since, including a multiple UAV variant^15^, or consideration of some operational restrictions^17^, though very few have considered the full extent of real world practicalities in their modelling process, particularly in a healthcare context, where factors such as packaging, payload size, routing, and timing restrictions may apply. A comprehensive survey of these issues has been captured by Thibbotuwawa et al.^16^.

With these oversimplifications in mind, this paper aims to investigate the realistic cost of using UAVs to enhance transit times in a mixed-mode logistics system, understanding how UAVs can best complement a traditional logistics service under real-world conditions. The paper also addresses knowledge gaps through answering the following research questions:

1. How does the relationship between transit time and operating cost affect delivery operations and modal selections?
2. How does site suitability impact the likely uptake of UAVs in a multi-modal delivery operation?
3. In what context would a multi-modal delivery service with UAVs offer tangible benefits?

## Methodology and Data

**Fig. 1:**
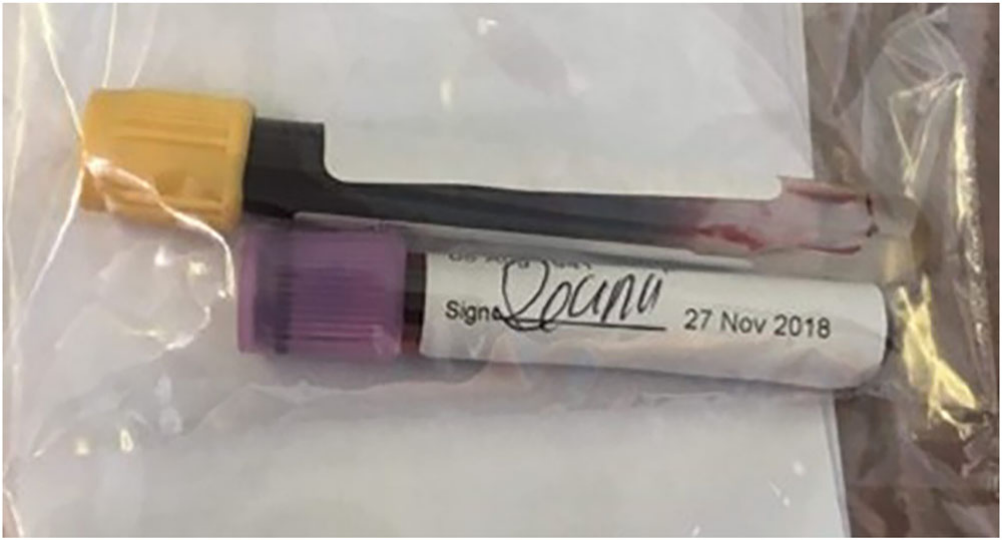
Typical diagnostic sample. Samples can vary in size but are usually a 5–10 cm long x 1–3 cm diameter.

An estimated 70–80% of medical decisions depend on diagnostic analyses of patient pathology specimens^67^, with typical day-to-day operations requiring the specimens that have been taken by healthcare staff at community clinics (e.g., Fig. 1) to be transported by van to nearby pathology laboratories usually located at hospitals for analysis.

Transport is often carried out by a third-party carrier with transit, delivery and collection times stipulated by the healthcare provider as part of the contract terms^68,69^. In an investigation of specimen collection optimisation, McDonald^70^ noted a healthcare practitioner preference for transit times of 90 min, whilst other studies have also imposed similar limits when investigating systems in other countries^71^, typically to maintain the quality of different samples^72^. To this end, this investigation tests the impact of constraining transit times to 90 min when compared to the current case study operating standard of 4 h (Southampton and IOW areas^73^), in addition to understanding the relationship between cost and transit time.

**Fig. 2:**
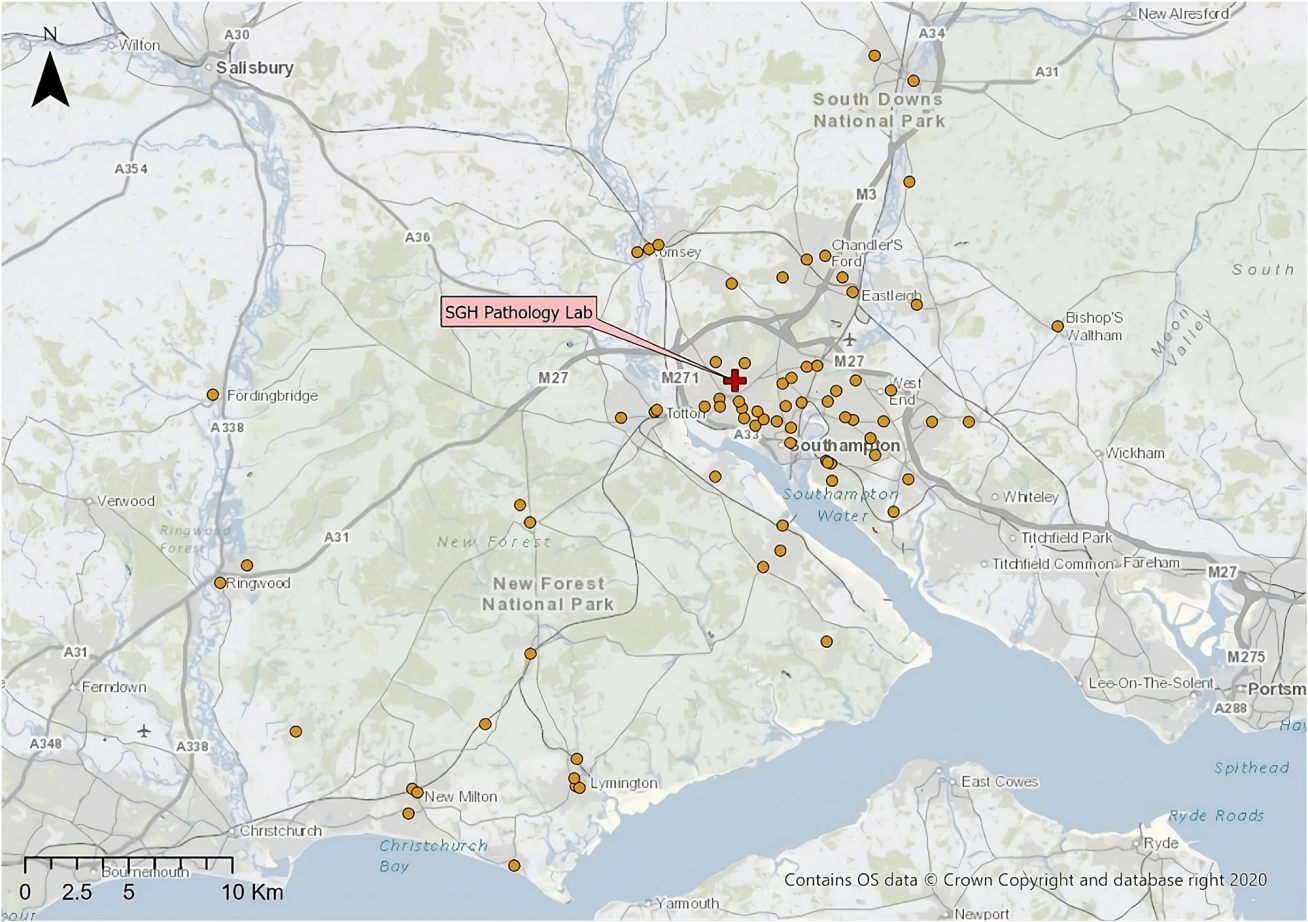
Southampton case study region map. SGH Southampton General Hospital (delivery point). Orange circles indicate community clinics. (Base Map © OpenStreetMap contributors).

**Fig. 3:**
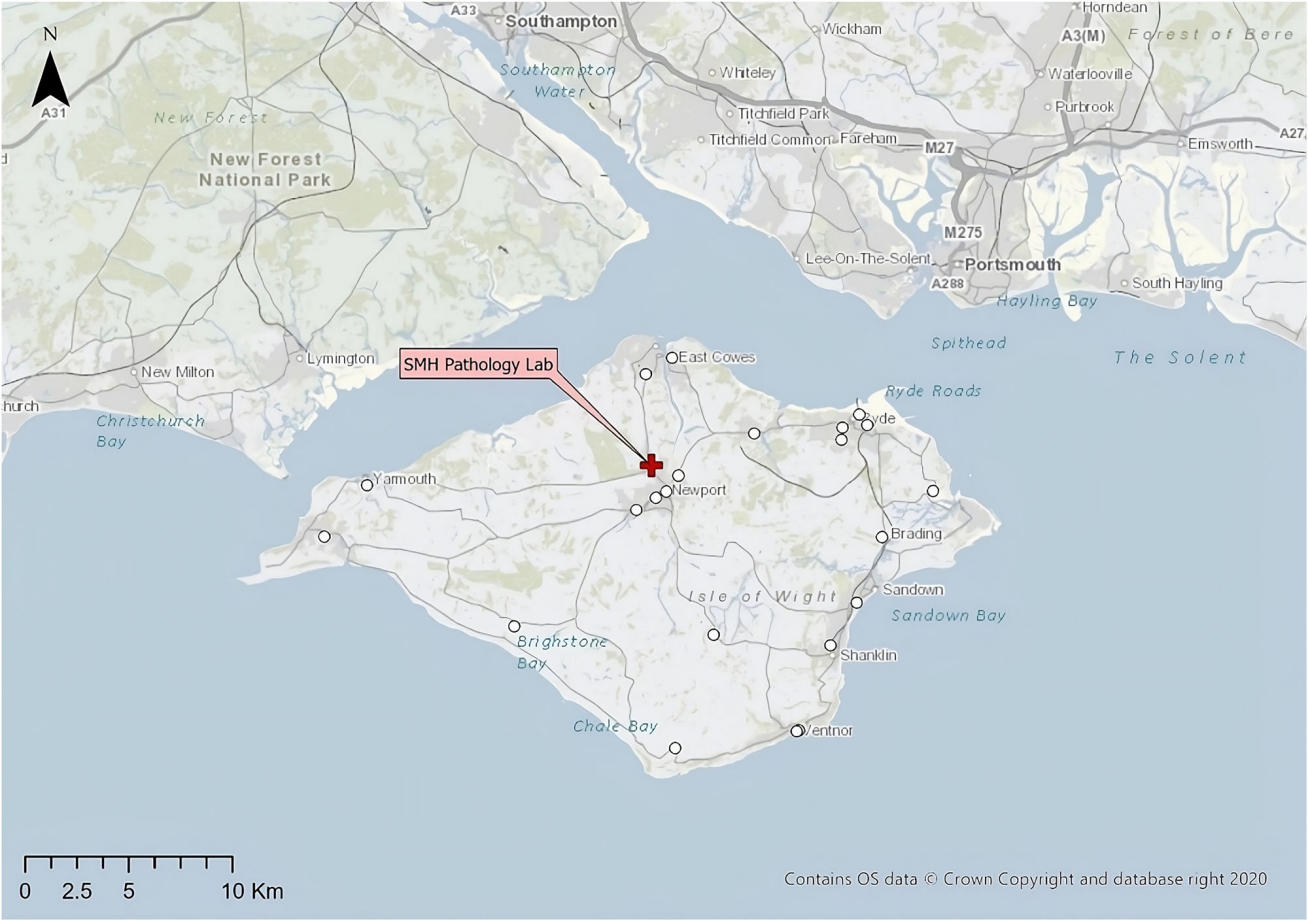
Isle of Wight (IOW) case study region map. SMH = Saint Mary’s Hospital (delivery point). White circles indicate community clinics. (Base Map © OpenStreetMap contributors).

**Fig. 4:**
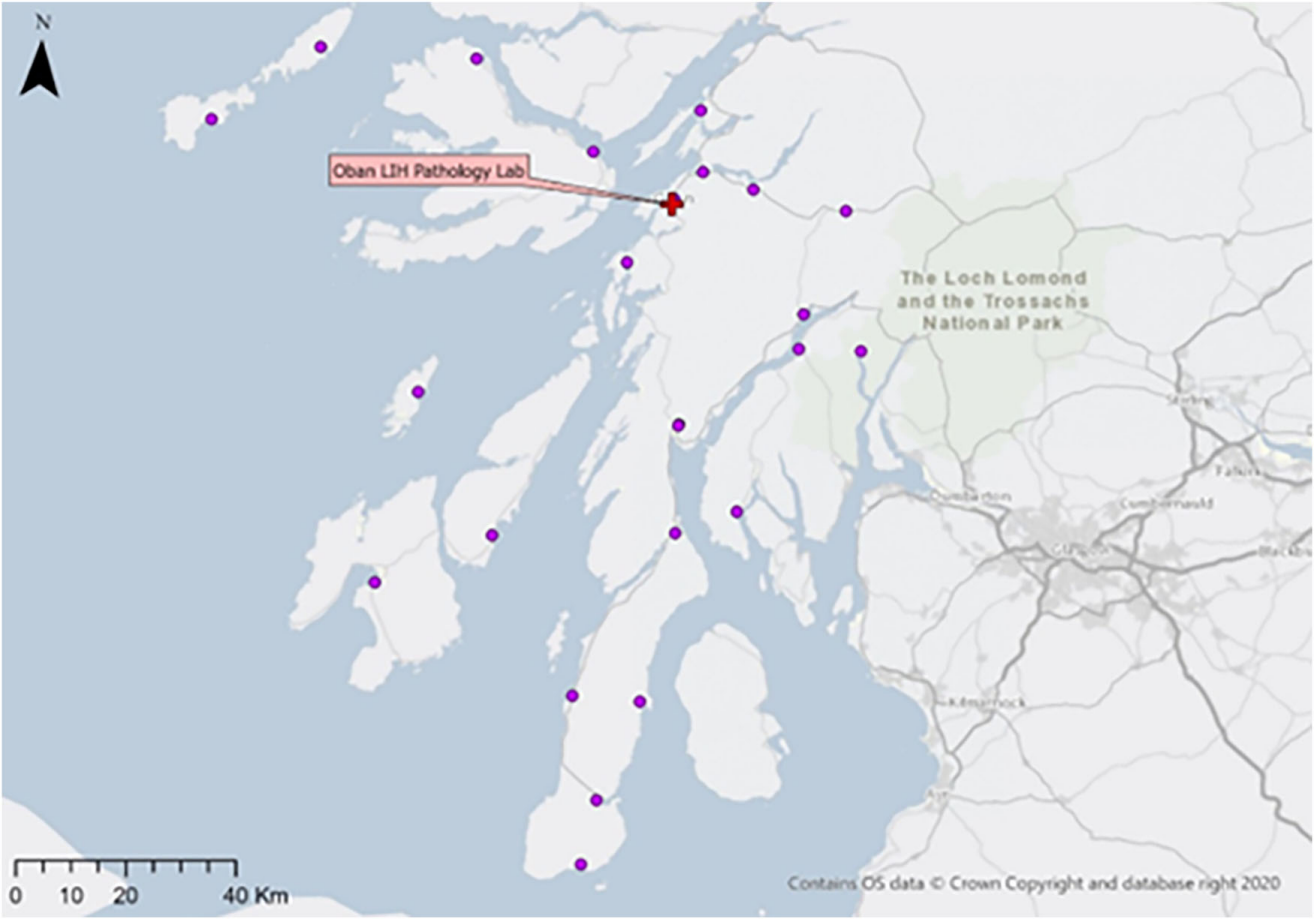
Hebrides case study region map. LIH Lorn and Islands Hospital (delivery point). Purple circles indicate community clinics. (Base Map © OpenStreetMap contributors).

This study uses three real-world case studies of diagnostic specimen transport to investigate the issues of transit time and transport equity:

1. **Southampton, UK**: a network of 76 community healthcare clinics distributed across the urban and peri-urban areas surrounding the city, serving a population of c.250,000 people (Fig. 2). Some 3100 samples are produced in a typical day within this region, before being transported to the laboratory at Southampton General Hospital (SGH) in the city of Southampton for analysis.
2. **Isle of Wight (IOW), UK**: a network of 22 community healthcare clinics distributed across the smaller and more rural areas on the island off the south coast of the UK, serving a population of c.25,000 people (Fig. 3). Approximately 330 samples are produced on a daily basis within this region, before being transported to the laboratory at St. Mary’s Hospital (SMH) in the small urban area of Newport.
3. **Hebrides, UK**: a network of 23 community healthcare clinics distributed across the very remote islands and mainland highland area on the west coast of Scotland in the UK (Fig. 4). The exact population covered by this catchment area is not known, but specimens are transported to the laboratory at Lorn and Islands Hospital (LIH) located in the small urban area of Oban, which has a population of c.8500. The sample production rate is also not known, though it is understood to be very small; however, all clinic sites still require a collection service.

The Hebrides case study also involved additional, location-specific transport arrangements for servicing clinics located on some islands (ferries and locally arranged car transport). It was not possible to capture these bespoke arrangements within the modelling tool directly; thus, manual adjustments were made post-modelling to capture these modes where they offered a competitive advantage over the selected solutions. For the manual calculations, travel distances and times were captured from Google Maps, whilst energy consumptions were captured using energy consumption factors assumed to be: (i) 0.6 MJ/km for a local e-van obtained from the manufacturer’s specification (Vauxhall Combo Electric van); and (ii) 161.0 MJ/km for a modern diesel-electric ferry^74^. As public transport, ferries would be traveling anyway, and therefore the energy consumed by carrying a container of specimens was allocated according to container mass (5 kg) as a fraction of a 50% laden ferry payload (100,850 kg for passengers, and vehicles combined), resulting in a factor of (5/100,850 × 161.0 = ) 0.008 MJ/km. Costs were based on cost factors assumed to be (i) £0.45 per mile (£0.72 per km), based on UK tax-free mileage rates^75^; and (ii) £2.40 per journey, based on the ferry operator’s small freight rates^76^.

**Fig. 5:**
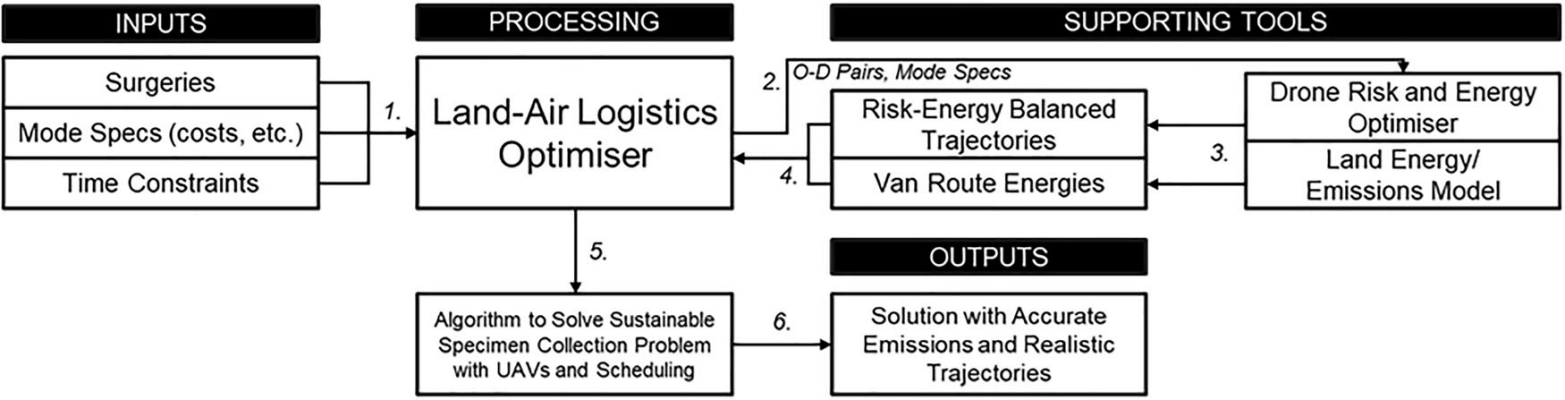
FORSETI modelling data flow. Interactions between the land-air logistics optimisation tool developed in this research and the supporting tools, collectively known as FORSETI. OD Origin-Destination. Numbers indicate the steps in the FORSETI model.

A modelling suite, known as FORSETI (Freight Optimisation with RiSk, Energy, and multi-mode Transport Integration), was used for analysing intervention scenarios based on realistic assumptions and data around UAV and ground logistics vehicle performance. FORSETI, first discussed and demonstrated by Oakey^64^ and Grote et al.^10^ respectively comprises multiple sub-modules which calculate: (i) UAV trajectories which are balanced between energy consumption^77^ and third-party ground risk^78^; (ii) ground vehicle travel times, distances, energy consumptions and emissions^79^; and (iii) a multi-mode optimisation tool which takes the origin-destination (OD) pair data captured in (i) and (ii), and produces a logistics plan which is optimised towards a given objective, using electric vans (eVans, electric light goods vehicle <4.25 T), cargo bikes, and UAVs^20^ (Fig. 5). Further details of each module are given in the remainder of this section, though full details of (ii) have been omitted for brevity due to the energy and emissions of the system not being the focus of this investigation.

To fully understand the impacts and sensitivity between transit times and costs with respect to the introduction of UAVs in a multi-modal transport network, several scenarios were explored for each case study region (Table 1). A baseline case was established for all regions, representing an idealised pseudo business-as-usual state that may be realised in the near future, using e-Vans for all collections (plus the local connecting modes within the Hebrides case study). A baseline scenario was used in place of the present-day business-as-usual position due to limitations around capturing the exact logistics operations from the providers involved, particularly in the Hebrides case, where discussions with local healthcare staff highlighted that current operations are notably inconsistent and depend on numerous, often unofficial, local arrangements, beyond those that have been captured (e.g., transport arranged by members of the public).

**Table 1:** Characteristics of the different scenarios investigated

Scenario ID	Time	Duration (h:m)	Path Lab	Number of Clinics	Number of UAV Permitted Clinics (% of total)	Maximum ITT(minutes)
Soton_Baseline	09:00–13:00	4:00	SGH	76	0 (0%)	240
Soton_Select_90	09:00–13:00	4:00	SGH	76	9 (12%)	90
Soton_Select_240	09:00–13:00	4:00	SGH	76	9 (12%)	240
Soton_All_90	09:00–13:00	4:00	SGH	76	76 (100%)	90
Soton_All_240	09:00–13:00	4:00	SGH	76	76 (100%)	240
IOW_Baseline	09:00–13:00	4:00	SMH	22	0 (0%)	240
IOW_Select_90	09:00–13:00	4:00	SMH	22	12 (55%)	90
IOW_Select_240	09:00–13:00	4:00	SMH	22	12 (55%)	240
IOW_All_90	09:00–13:00	4:00	SMH	22	22 (100%)	90
IOW_All_240	09:00–13:00	4:00	SMH	22	22 (100%)	240
Heb_Baseline	09:00–17:00	8:00	OBN	23	0 (0%)	480
Heb_All_480	09:00–17:00	8:00	OBN	23	23 (100%)	480
Heb_UAVx_480	09:00–17:00	8:00	OBN	23	23 (100%)	480

*SGH* Southampton General Hospital, *Soton* Southampton, *IOW* Isle of Wight, *SMH* St Mary’s Hospital, *LIH* Lorn and Islands Hospital, *ITT* In Transit Time.

Within the Southampton and IOW regions, four further cases were explored: (i) where UAVs are only permitted to visit sites where there is sufficient landing space, with (a) a 90-min transit time limit imposed, and (b) the current transit time limit of 4 h (240 min) imposed; and (ii) where UAVs are permitted to visit any site in the case study area, also with (a) a 90-min transit time limit imposed, and (b) the current transit time limit of 4 h (240 min) imposed. These tests were also conducted with the relative importance of cost and transit time being varied between cost optimised and transit time optimised (see Section “Ground Logistics Mode Characteristics and Routing”), exploring the sensitivity between these two objectives. The impact of site suitability and transit time limits on this relationship were also explored.

Owing to the need for manual post-processing, only two additional scenarios were explored for the Hebrides case, where results were (i) optimised towards minimising transit time only; and (ii) based on having UAVs as the only mode input, to enable a comparison of a fully UAV served system against a time-optimal system. An 8 h transit time limit was imposed (equal to the length of the shift period, see below), and no cycle couriers were permitted due to the scale of the area in question (e.g., maximum range of 8 km, Section “Ground Logistics Mode Characteristics and Routing”) and the likely unavailability of a cycle provider in the area.

The historic data also considered a full day of operation, which can be divided into two distinct periods (morning and afternoon). To simplify comparisons, the established baseline solutions and tests described in this paper modelled a single collection shift, with each study including all the sites from the original dataset. In the Southampton and IOW cases, this was a morning shift (09:00–13:00), whilst the Hebrides was a full day (09:00–17:00) due to the size of the area and small number of carried samples. Additionally, a 2.5 min dwell time was assumed for all collections and deliveries^80^ and reverse logistics, such as the replenishment of empty containers, was assumed to be completed as part of the collection system.

### Logistics model

In combination with cost parameters and time constraints, the logistics optimisation element of FORSETI takes a list of clinics requiring service in a given shift period (e.g., morning/afternoon) as an input, before using the relevant OD data from the other modules (see Sections “UAV Mode Characteristics and Routing” and “Ground Logistics Mode Characteristics and Routing”) to produce a logistics plan to meet demand, considering multiple vehicle modes. In this investigation, vans and UAVs operated from the target hospital, whilst cyclists could be based at any of the sites in the input list.

**Fig. 6:**
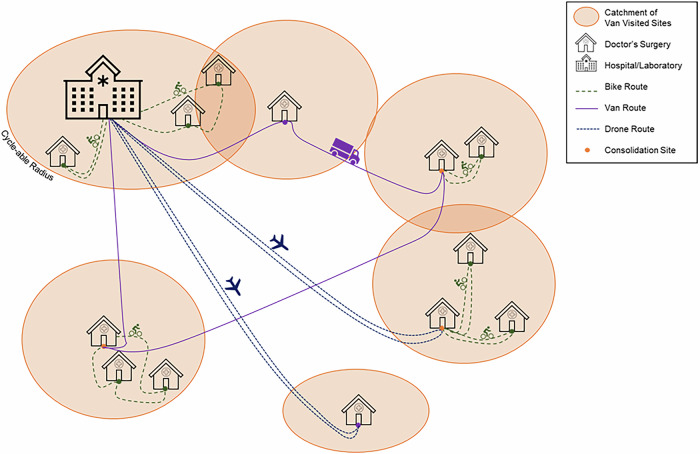
Proposed routing options in the proposed multi-modal healthcare logistics system arrangement. Cyclists consolidate in local areas or to the hospital, whilst UAVs and vans complete the intermediate collections over longer distances.

Regarding route structures (Fig. 6), cyclists were permitted to carry loads from up to three surgeries to either consolidate loads on a local basis or deliver directly to the hospital. Vans could collect from these consolidation sites or any other sites with no limitation on the number of stops per route. UAVs could serve any permitted site (see Section “UAV Mode Characteristics and Routing”) and only had operating capacity for a single stop before returning to the hospital for delivery, assuming that any cycle consolidated loads are transferred to a single container.

Building on the business-as-usual cases, where each site generally receives 2 collections per day (1x morning, 1x afternoon), each surgery was served once in a given shift period. For simplicity, only 1x shift period (morning, 09:00–13:00) has been modelled, under the assumption that similar trends would occur during the other shift (afternoon).

For brevity, the full formulation of FORSETI’s logistics module has been omitted, though the objective function has been given below (Equation 1). It aims to minimise the weighted sum of: (i) the component costs of operating the logistics system, including any labour (e.g., drivers), vehicle running costs (e.g., fuel, etc.), and vehicle standing costs (e.g., servicing, insurance); (ii) the priced environmental impact, derived from the energy associated with operating each route; and (iii) the maximum in-transit time across all routes. The optimisation algorithm deployed in this module is an adaptation of the well-established and efficient Clarke and Wright Savings Algorithm combined with bin-packing algorithms, accounting for multiple modes, multiple-legs, and shift planning. The full description, formulation and pseudo-code of the logistics planning module can be found in Oakey et al.^20^.

Equation 1. FORSETI objective function.

$$\min :\sum _{{\bar{r}}_{k}\in \bar{R}}\left({x}_{{\bar{r}}_{k}}\left(\sum _{{r}_{v,k}\in {\bar{r}}_{k}\cap {R}^{V}}{{\rm{\theta }}}_{1}{p}_{{r}_{v,k}}+\sum _{{r}_{d,k}\in {\bar{r}}_{k}\cap {R}^{D}}{{\rm{\theta }}}_{2}{p}_{{r}_{d,k}}+\sum _{{r}_{c,k}\in {\bar{r}}_{k}\cap {R}^{C}}{{\rm{\theta }}}_{3}{p}_{{r}_{c,k}}+{{\rm{\theta }}}_{4}{{\rm{\epsilon }}}_{{\bar{r}}_{k}}{\rm{\gamma }}\right)\right)$$

$$+{{\rm{\theta }}}_{1}{W}^{V}{A}_{\max }^{V}+{{\rm{\theta }}}_{2}\left({W}^{D}{A}_{\max }^{D}+{W}^{O}{A}_{\max }^{O}\right)+{{\rm{\theta }}}_{5}u$$

In the optimisation process, collection rounds (${\bar{r}}_{k}\in \bar{R}$) are created to combine a van or UAV (**d**rone) route (${r}_{v,k}$ or ${r}_{d,k}$, respectively) with a set of consolidation bicycle courier routes (${r}_{c,k}$). Each route has an associated cost (${p}_{{r}_{v,k}}$,$\,{p}_{{r}_{d,k}}$,$\,{p}_{{r}_{c,k}}$) that is a function of its distance and duration. Total labour costs for each shift period are given by a function of the maximum number of drivers, UAVs, and UAV operators required at any point (${W}^{V}{A}_{\max }^{V}$ for vans, ${W}^{D}{A}_{\max }^{D}$ for UAVs, and ${W}^{O}{A}_{\max }^{O}$ for UAV operators). Cyclist labour is included in the route costs due to being sourced on an ad-hoc basis using a gig-economy courier arrangement.

The emissions of a collection round are denoted by ${{\rm{\epsilon }}}_{{\bar{r}}_{k}}{\rm{\gamma }}$ and are calculated as a product of the energy consumed and a constant emissions factor per unit of energy. In this paper, the emissions factor was taken from the UK Government’s published factors that are representative of the typical UK electricity network mix, accounting for the lifecycle of the electricity, from production and transmission, through to end use, at a total fixed rate of 0.2439 kg CO_2_-eq/kWh (used for all electric modes). The energy consumptions for vans and UAVs were calculated as part of the other modules within FORSETI. Whilst emissions were not the focus of this investigation, it should be noted that should the electricity grid’s mix change (e.g., future increase in electricity generated from renewable sources), then the emission factors would also change.

Ferries were diesel-electric, generating energy from on-board diesel generators. Therefore, emissions were calculated manually by converting the energy consumed to diesel combusted based on diesel’s net calorific value (42.7 MJ/kg) and density (840 kg/m3), and then multiplying by the UK Government’s published emissions factor for diesel (lifecycle emissions of 3.1677 kg CO_2_-eq/litre). If ferries become fully electrified, it would be possible to use the same emissions factor as for all other electric modes (i.e., 0.2439 kg CO_2_-eq/kWh) instead.

The transit time metric ($u$) is calculated as the maximum transit time between collection and delivery across all rounds and collections within the system. For sites that are served via a consolidation route, the duration from first departure from the origin site is used. It should be noted that this metric was selected over alternatives such as the average transit time across all rounds to ensure that care standards remained equitable, similar to how emergency services such as non-emergency ambulances are planned and measured, catering to the 90^th^-percentile^81^. Using an average value risks a skewed distribution of transit times that may favour certain geographical areas. To limit this variable under some scenarios, a constraint (${{t}_{\bar{r}}}^{\max }$) can be applied, where collection round transit times must not exceed the value of ${{t}_{\bar{r}}}^{\max }$.

The optimisation used a weighted objective function to facilitate balancing each objective and their relative importance (as demonstrated by Grote et al.^10^), where each objective (cost, emissions, and transit time) is somewhat conflicting in a three-way trade-off. In the case of this study, the weighted sum was configured such that the theta values (${{\rm{\theta }}}_{1}$ to ${{\rm{\theta }}}_{5}$) isolated the relationship between total operating cost and transit time; hence, ${{\rm{\theta }}}_{4}$ (energy/emissions) = 0. A parameter sweep was conducted, varying the relative importance of ${{\rm{\theta }}}_{5}$ (transit time) compared to fixed ${{\rm{\theta }}}_{1}$, ${{\rm{\theta }}}_{2}$ and ${{\rm{\theta }}}_{3}$ (van, UAV and bicycle courier costs, respectively), enabling a Pareto front of solutions to be found with respect to these objectives. It should be noted that cost assumption sensitivities have been explored as part of a separate study and are beyond the scope of this investigation^10^, and an optimistic position was adopted to enable a representative comparison in this study. Emissions sensitivities were inconsequential to the results due to the zero-weighting on the objective function.

For each case study area, the demand inputs used in FORSETI contained a list of clinics that required service (postcode locations) within the shift period. For complete coverage, all of the sites featured in the case study descriptions were included in all model runs. Additionally, it should be highlighted that the baseline positions established for each case study using FORSETI ensured that the impacts of any intervention were evaluated operating under the same assumptions, e.g., the same site dwell times; thus, reducing any systematic errors that may occur as a result. The intervention results were subsequently compared to this benchmark to prevent overstating the benefits/disbenefits of a given solution.

### UAV mode characteristics and routing

UAV trajectories were planned using a combination of two sub-models, resulting in trajectories that were optimised as a reasonable balance of energy consumption and third-party ground risk (i.e., the safety of those who are exposed to drone flight on the ground). Ground risk was estimated in terms of fatalities per journey, based on the average (mean) risk of a fatality due to the UAV crashing on its flight path resulting from a failure that renders it no-longer able to remain airborne (e.g., electronic failure). The probability of colliding with a third-party with sufficient energy to cause a fatality was calculated using UAV descent models (ballistic/glide/parachute) and population mapping (from census and land-use data), which varies throughout the day. Pilko et al.^78^ details a full description, including the numerical formulation.

Ground risk minimising UAV trajectories generally avoid built-up areas and can often take circuitous (indirect) routes to do this^82^; meanwhile, energy optimal trajectories will follow a straight line^77^. Even though many UAV logistics studies also assume straight line flights^16^, practical routes in future applications will likely require a balance of these two strategies, meaning routes will have at least some level of circuity. To this end, FORSETI combines both aspects when developing UAV trajectories, creating a path, duration, and distance for each UAV-suitable OD pair.

Within the logistics model, UAVs are permitted to complete single stop trips due to operational and capacity constraints; however, they are able to carry loads that have been consolidated locally by cycle routes. All UAV routes start and end at the destination hospital, where battery swaps are assumed to take place, requiring an additional 10 min of dwell time for each UAV prior to completing any additional journeys beyond the first trip (5 min for the battery swap + 5 min for a pre-departure airworthiness check^83^). Under this arrangement, it has been further assumed that the UAVs are not range limited within the limits of the case study areas and can serve all sites without further battery changes or recharging if they are permitted to do so. Wind effects were assumed to be negligible due to UAVs following the same flight path on the outbound and return legs of collections, such that prevailing tailwinds become headwinds on the reverse leg.

**Fig. 7:**
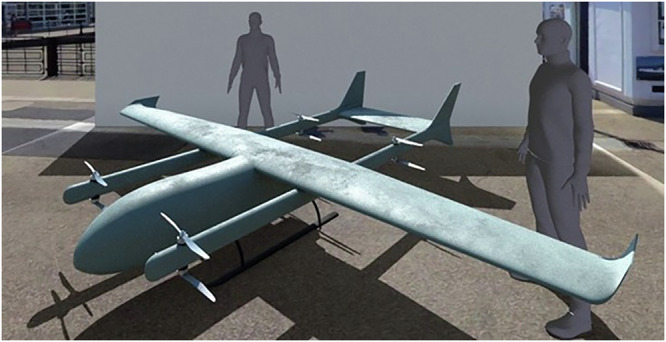
Render of modelled 5-metre wingspan UAV platform. Payload positioned in main body (one industry standard medical container (dimensions 460 × 255 × 305 mm, see Fig. 8)).

**Fig. 8:**
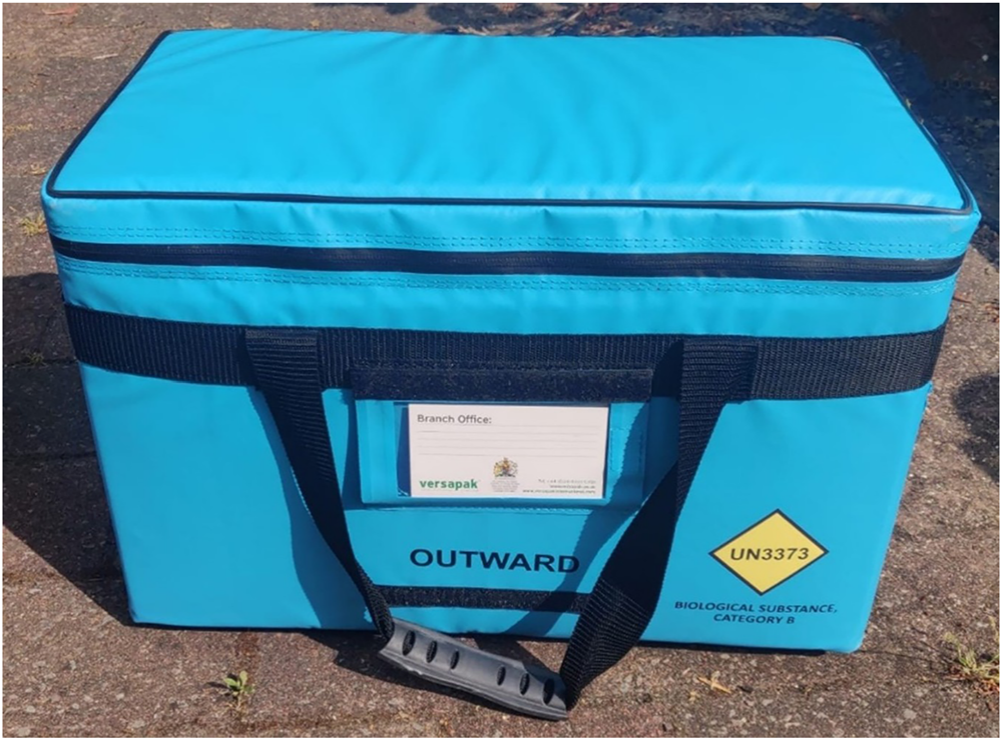
Industry standard insulated medical container (brand name Versapak). Typical dimensions 460 × 255 × 305 mm, empty mass 2.2 kg, mass when fully loaded with specimens ~5 kg^123^.

To adequately accommodate the payload requirements, a UAV of sufficient size and power is required. Vertical Take-Off/Landing (VTOL) capabilities are also a key requirement due to the limited scope for conventional take-off/landing at all sites, whilst a hybrid VTOL-fixed-wing platform may be required to ensure sufficient range and improved in-flight performance in many cases. Additionally, the platform must conform to Dangerous Goods operating conditions in line with conditions set by the national aviation authority^2^. To this end, this investigation simulates the use of an electric power fixed-wing/VTOL hybrid UAV with a wingspan of 5 metres (Fig. 7), similar to one used in real-world medical delivery trials^84^, capable of carrying one industry standard medical package (Fig. 8), with an estimated range of 100 km and assumed cruise speed of 65 km/h. The choice of UAV was based on practical trials undertaken by the authors as part of the EPSRC funded e-Drone (www.e-drone.org) and DfT funded Future Transport Zone (www.solent-transport.com/drones/) projects.

Whilst not considered within the objectives, the reported emissions were calculated using the energy estimation methodology detailed by Blakesley et al.^77^, based on the characteristics of a known electrically powered multi-copter platform with a similar Maximum Take-Off Mass (MTOM) and carrying capacity but considerably shorter range. Calculations determined energy consumption using a physics-based, instantaneous energy consumption model (i.e., varying with a drone’s phase of flight such as take-off, climb, cruise, descent, landing), with characteristics based on commercially available drones, and the estimations were subsequently scaled by half to account for the energy consumption differences seen in fixed-wing UAVs vs. copter-type UAVs^85^.

When using such a large UAV, sites that can be practically and safely visited may be limited if landing is a requirement; thus, an audit of the case study sites was conducted using satellite mapping, as presented by Oakey et al.^12^. Surgeries were assumed to be suitable if they had a suitable UAV landing area on site or nearby, e.g., an area of open space of approx. 100 m^2^ within the site grounds or on public land just outside, reducing the need for onward connections and the introduction of challenges associated with the “final 50-feet”^86^. This assumption is a slight deviation from current UK aviation standards, on the basis that rules may be slightly relaxed for trained operators in future^87,88^. To understand the impact of these restrictions, a comparison has been drawn between scenarios with restricted site suitability, and those permitting all sites for service through use of developed collection systems (e.g., winch systems or reception facilities^89^). Given the rural nature (i.e., unbuilt-up) of the areas around the Scottish Hebrides sites, it was assumed that UAVs would be permitted to serve all of the sites in the Hebrides case.

With respect to UAV cost parameters (Table 2), the inputs were derived from Oakey^64^ and Grote et al.^10^, where a theoretical future scenario is applied, assuming increased levels of automation, manufacturing cost reductions, and relaxed operating legislation. Staff costs (£31.44 /hr) accounted for mission commanders overseeing operations at a ratio of 1 operator per 20 UAVs in line with recently emerging real-world parameters for large scale operators^90,91^; and a loader/technician based at the hospital, responsible for payload management and continued airworthiness checks (in line with typical aviation standards^92^. Vehicle costs (£20.33 /hr) accounted for the purchase and maintenance of the components (based on expected life expectancies) and electricity consumption. A daily standing cost of £8.99 /UAV/day was also applied to account for operational insurance and airspace management fees^93^.

**Table 2:** Cost configurations used for each transport mode during modelling (2022/23 prices)

Description	Units	Van	UAV	Bicycle Courier
Vehicle running cost per unit distance	£/mi (£/km)	0.34 (0.55)	-	1.01^a^ (1.63)
Labour cost per hour	£/hr	11.93	31.44	-
Vehicle running cost per hour	£/hr	-	20.33	-
Cost per task	£/task	-	-	7.07
Cost per stop in addition to the first stop	£/stop	-	-	2.78
Vehicle standing cost per day	£/veh/day	29.33	8.99	-
Maximum pickups/stops per return trip	Stops	No Limit	1	3
Maximum permitted range ^b^	mi (km)	78 (125)	62 (100)	5 (8)

^a^Beyond a threshold distance of 0.5 mi. ^b^Relaxed for Hebrides case study.

### Ground logistics mode characteristics and routing

With respect to the ground vehicle data, FORSETI captures the OD travel data (distance, time) from the Google API using the “realistic” travel speed option, taking traffic into account. To this end, the eVan data in this study were obtained using one-hour intervals across the modelled period, assuming constant conditions for each hour to save on data capture requirements (e.g., 09:00 data are assumed to apply 09:00–10:00). For cycling, it was assumed that travel times were unaffected by traffic, meaning the data were assumed to be constant for the full analysis period. Additional calculations covering OD travel energies and emissions for eVans are also calculated within FORSETI^79^, though these were not included in this study’s objective function due to travel times being the focus. Risk estimates were not included in the calculation of van routes due to routes being constrained to existing road network, unlike UAVs, where flight paths are not as restricted.

For compliance with practical limitations, all solutions were checked to ensure the maximum number of medical containers carried by any van during any shift (either loaded with samples or empty for reverse logistics, Section “Logistics Model”) was < 20 to provide a buffer below the typical eVan payload capacities (~5 m^3^ and ~800 kg from manufacturers’ specifications). Additionally, travel distances were constrained to ensure the maximum distance travelled during a shift never exceeded 125 km; remaining within the limits of typical e-van range abilities (200+ km range quoted in manufacturers’ specifications). The range constraint had to be relaxed in the Hebrides area due to the distances involved (longest route in baseline = 412 km), meaning that intermediate charging stops may be required.

The cost parameters (Table 2) used within the van calculations were based on the Manager’s Guide to Distribution Costs published in the UK by the Freight Transport Association^94^, capturing full ownership costs such as maintenance and depreciation. In the absence of other comparable data, the cost values for diesel vans were used for eVans as a reasonable estimate, given eVans are now competitive for Total Cost of Ownership^95^, with initial purchase costs being higher, but day-to-day operational costs being lower to offset this difference. Labour costs for drivers (£11.93/h) were charged for a full shift period regardless of driving time, as may be expected for drivers working to a contract; vehicle running costs (£0.34/mi, £0.55/km) were charged per mile and included fuel (electricity), tyres, and maintenance; and vehicle standing costs (£29.33/vehicle/day) were charged per day and included vehicle tax, insurance, depreciation, and overheads.

Cycle couriers were costed according to the price structures at a real-world cycle courier company in the UK^96^. Fees (Table 2) are charged on a per task basis (£7.07/task), with each consolidation route being one task. One collection and 0.5 miles of travel distance is included in each route fee, and additional charges (£2.78/stop, £1.01/mi or £1.63/km) are incurred for each increment beyond these thresholds. Hence the total cost per cycle route can be calculated by: ${Cost}=7.07+2.78({n}_{c}-1)+1.01(d-0.5)$, where ${n}_{c}$ is the number of stops and $d$ is the route distance in miles. Cycles were considered to have capacity for up to three medical containers (see Fig. 8) and were limited to a maximum route length of 8 km to remain within typical limits for this mode^97^. Energy consumption and emissions were assumed to be zero for all cycle routes.

## Results

### Southampton

**Fig. 9:**
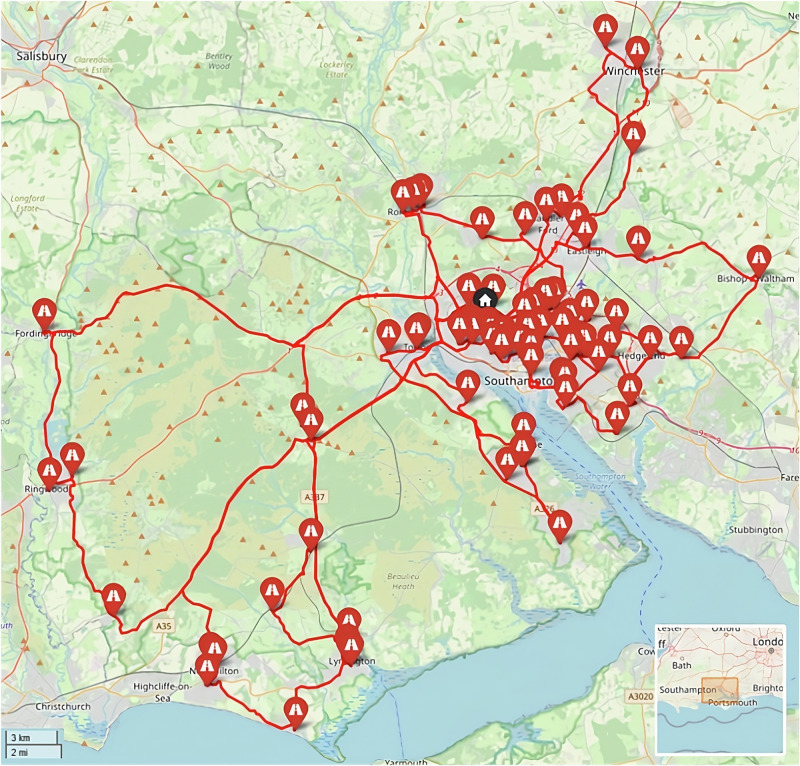
Example routing arrangement for the Southampton Baseline case (Soton_Baseline). Red lines indicate van routes. Black pin indicates the base/destination hospital. (Base Map © OpenStreetMap contributors).

The baseline case in Southampton (Fig. 9) uses 4 vans to complete all the collections from the 76 locations served in a typical morning shift period, travelling a total of 200 miles (325 km) in a total driving time of 13 h 24 min (including dwell). The cost was estimated to be £377 to cover all the vans and drivers used in the 4-hour shift period, equating to an annual cost of £189,900 (assuming 252 working weekdays and two collection runs per day). In terms of transit times, the maximum transit time (duration between first pickup in a round and delivery) was 209 min.

**Fig. 10:**
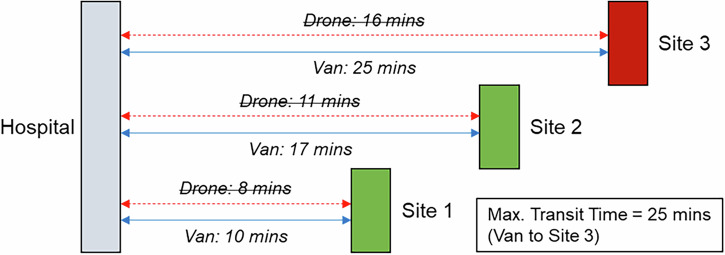
Example impact of optimising to minimise the maximum in-transit time when only select locations can be UAV served. Only Sites 1 and 2 are suitable for UAV service. Vans serve all sites.

Under the scenario where UAVs are only permitted to visit locations where there is sufficient space for landing safely (see Section 3), introducing UAV services did not offer any incremental transit time benefits, unless the site limiting the maximum transit time site was UAV serviceable. This is demonstrated in Fig. 10.

**Fig. 11:**
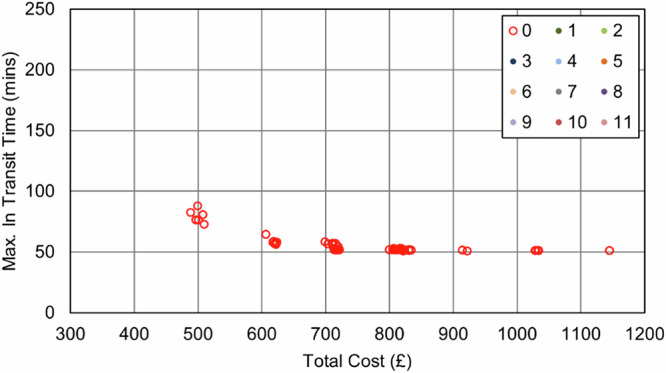
Soton_Suit_90 – Cost and Maximum Transit Time Trade Relationship. Point colours relate to the number of UAVs in each solution.

**Fig. 12:**
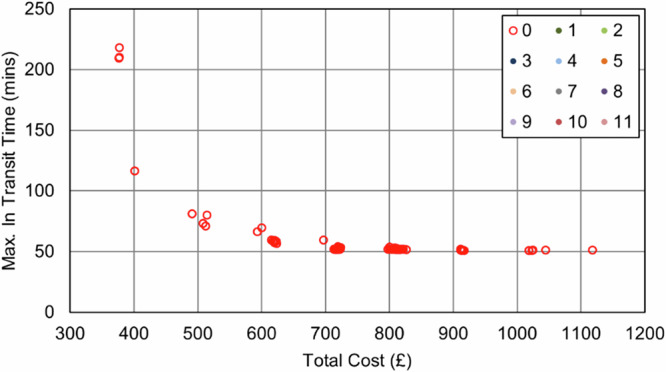
Soton_Suit_240 – Cost and Maximum Transit Time Trade Relationship. Point colours relate to the number of UAVs in each solution.

In the Southampton case specifically, increasing the importance of the maximum transit time objective (relative to the cost objective) resulted in an increase in van use only, with each van making fewer stops to reduce the time any consignment spends in transit. This pattern continued until the surgery that was furthest from the delivery point was served by its own dedicated route, effectively capping any further progress towards this objective, reducing the maximum transit time from 209 min (baseline) to 51 min (76% reduction, 8 vans, 0 UAV, 0 bike). As evidenced in the full parameter sweep results in Fig. 11 and Fig. 12, an improvement in transit time generally caused an increase in van use and resulting cost, with the maximum time reduction increasing costs by 119%.

UAVs were not selected in any balance of objectives due to the maximum transit time sites being unsuitable for UAV service, meaning any routes that could be substituted for UAVs would not offer any service increment when the whole area is considered. Similarly, bikes did not reduce the transit time due to the critical surgeries not having neighbouring surgeries close enough to consolidate and reduce the maximum time.

As expected, the impact of the transit time limit (${{t}_{\bar{r}}}^{\max }$) was only evident as transit times approached 90 min, with the Soton_Suit_90 solutions (Fig. 11) being capped at 90 min and a cost of £491 per shift period (5 vans, +31% cost over the baseline). With more flexible delivery timescales (240 min, Soton_Suit_240, Fig. 12) that allowed the use of longer, more stop-efficient routes, fewer vehicles were required, and costs decreased as a result. For example, the most cost-effective solution in this analysis was the enhanced business-as-usual case, where relaxing the transit time constraint to 240 min made a solution with 4 vans feasible, at a cost of £377 per shift period and a maximum transit time of 209 mins. It should also be highlighted that intermediate solutions were also identified between these two extremes that could offer a compromise to decision makers: for example, a 4-van solution costing £401 per shift period (+6% cost over the baseline), with a maximum transit time of 117 min.

**Fig. 13:**
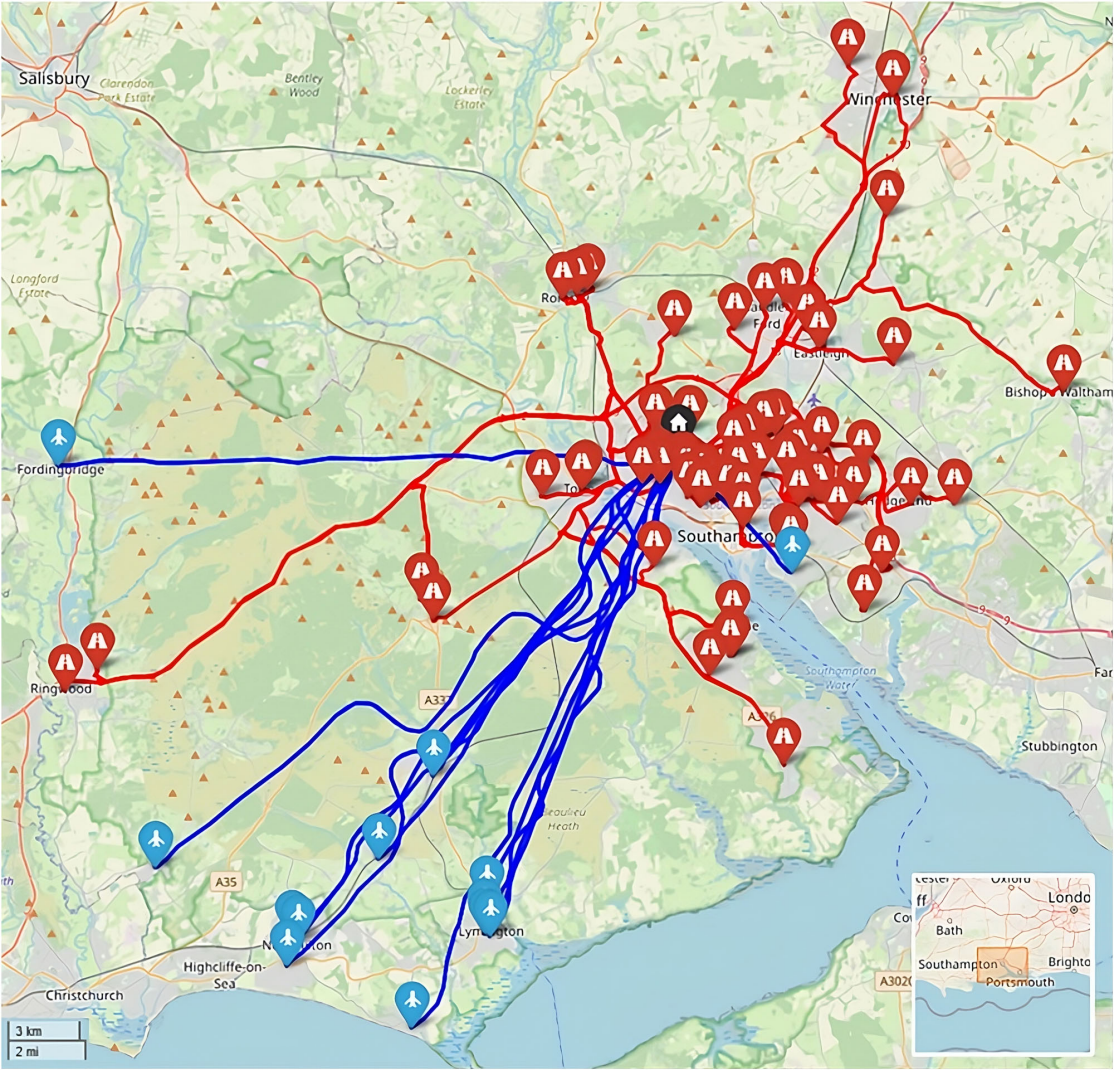
Example routing arrangement at maximum UAV uptake in the Soton_All Scenarios, i.e., UAVs to the furthest sites, vans to the majority of the remainder. Red lines indicate van routes, blue lines indicate UAV routes.

**Fig. 14:**
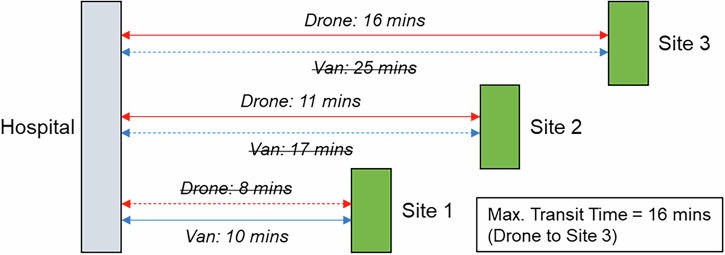
Example impact of optimising to minimise the maximum in-transit time when all locations can be UAV served. UAVs serve Sites 2 and 3, van serves Site 1.

Permitting all sites for UAV service (e.g., by using a winch delivery system or similar) results in a different outcome, where UAV uptake increases with the relative importance of the transit time objective (Fig. 13). All individual OD pair travel times between the hospital and surgeries were faster by UAV than by van, meaning the furthest sites are transferred to UAV service first. This pattern continues until the transit time of the longest UAV route prevents further progress towards the objective. At this point, the longest UAV route is longer than the longest van route and no improvement can be made whilst maintaining the principle of equitable care. This limit concept is illustrated in Fig. 14.

Throughout the parameter sweep, a maximum of 10 UAVs and 1 van were used in place of the 4 vans from the baseline case. Additionally, up to 3 bike tasks were also introduced to assist through consolidation routes. A maximum 84% reduction in transit time was made possible by using a multi-mode solution; however, costs increased by 133% in order to achieve this (1 van, 10 UAVs). On an annual basis (252 working weekdays, 2 shift periods per day), this would result in an additional £253.4k in operating costs and an additional 13.5 tonnes (+88%) of CO_2_-eq. emissions; substantial increases that would need to be accounted for by the NHS.

**Fig. 15:**
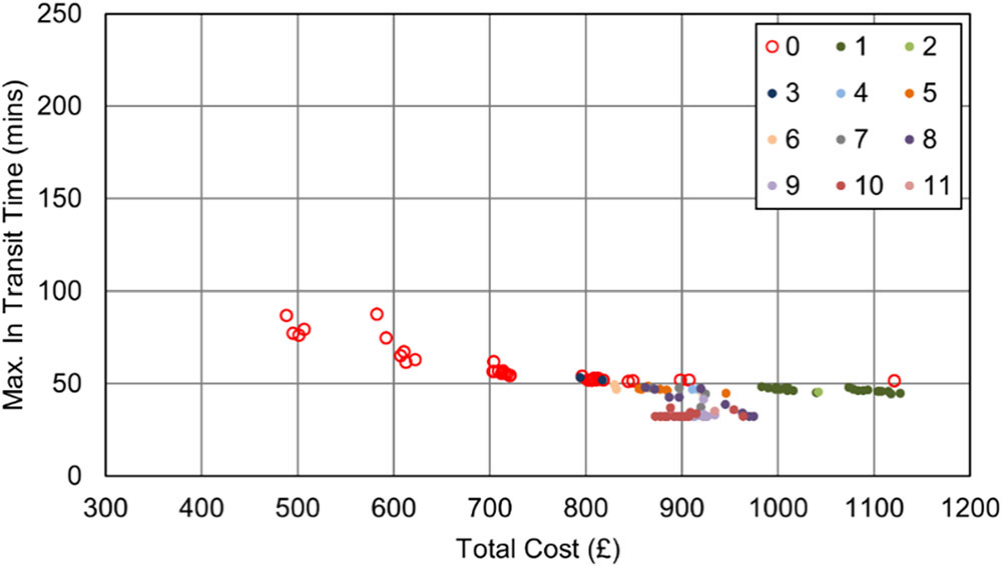
Soton_All_90 – Cost and Maximum Transit Time Trade Relationship. Point colours relate to the number of UAVs in each solution.

**Fig. 16:**
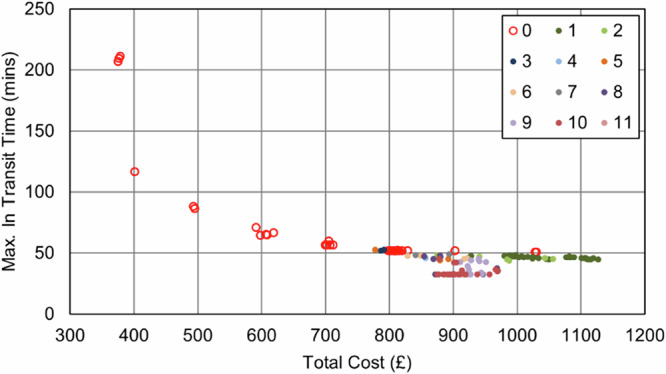
Soton_All_240 – Cost and Maximum Transit Time Trade Relationship. Point colours relate to the number of UAVs in each solution.

With respect to cost, the introduction of UAVs into the system results in a substantial increase, even as vans are removed from the system. This is due to vans offering an economy of scale by avoiding additional stem-mileage, unlike with UAVs, where each collection requires an independent trip. After the initial uptake in UAVs, there is some benefit offered by increased service to some sites, with additional short journeys being less financially damaging if they can help to eliminate vans and their associated costs. Given the fixed UAV costs (platform and operator) are charged regardless of utility, it is therefore advantageous to “sweat the asset” and increase the utility of any UAVs used. This cost relationship is seen in both Fig. 15 and Fig. 16, where the plot shows an increase in cost with UAV introduction, but then minimal increases for further transit time improvements beyond this.

It should be noted that in both of the all sites permitted scenarios in Southampton (i.e., Soton_All_90 and Soton_All_240), no solution contained solely UAVs, due to vans being capable of serving those sites nearest to the hospital as fast/faster than UAVs could serve the sites furthest from the hospital. Furthermore, all solutions where UAVs were chosen resulted in an increase in costs relative to the baseline case.

### Isle of wight

**Fig. 17:**
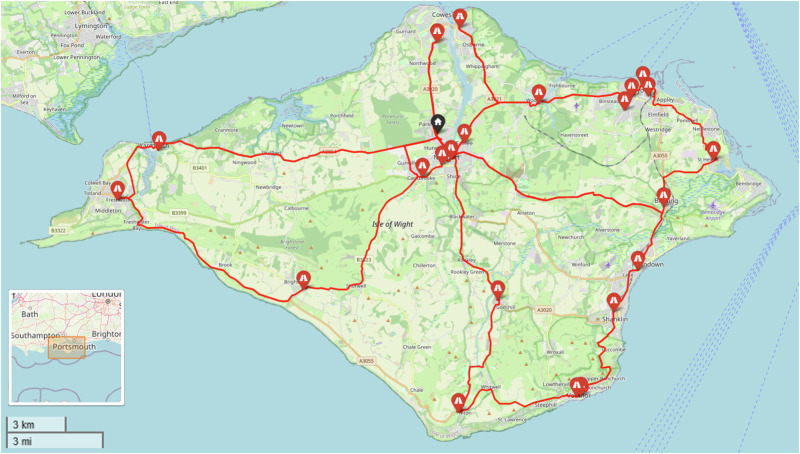
Example routing arrangement for the IOW Baseline (IOW_Baseline) case. Red lines indicate van routes. Black pin indicates the base/destination hospital. (Base Map © OpenStreetMap contributors).

The baseline case in the IOW (Fig. 17) uses 2 vans to visit the 22 locations served in a typical morning shift period and complete all collections. Travelling a total of 86 miles (138 km) with a total driving time of 4 h 50 min (including dwell), the cost was estimated to be £183 to cover all the vans and drivers used in the 4-hour shift period, equating to an annual cost of £92,300. With respect to transit times, the maximum transit time (duration between first pickup in a round and delivery) was 198 min.

**Fig. 18:**
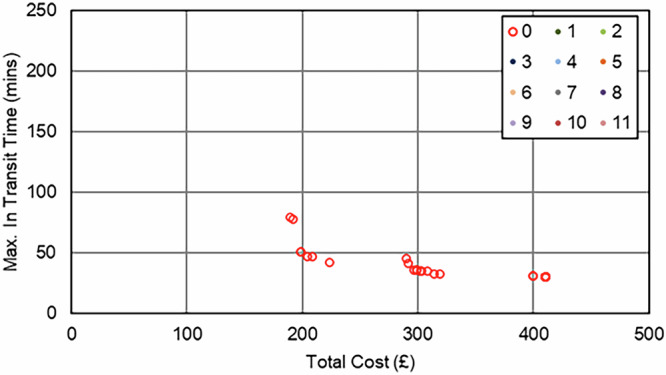
IOW_Suit_90 – Cost and Maximum Transit Time Trade Relationship. Point colours relate to the number of UAVs in each solution.

**Fig. 19:**
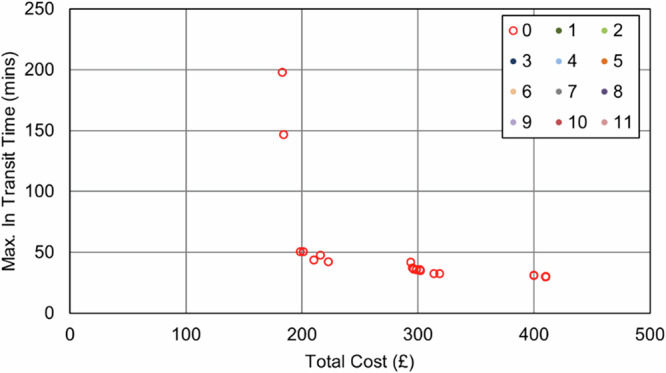
IOW_Suit_240 – Cost and Maximum Transit Time Trade Relationship. Point colours relate to the number of UAVs in each solution.

As was the case in the Soton_Suit scenarios, when UAVs were only permitted to serve those sites that were suitable for a safe landing, no UAVs were selected. The number of stops in each van route decreased to reduce the maximum transit time, introducing additional vehicles and cost into the process (Fig. 18 and Fig. 19). The best transit time achieved was 30 min (84% reduction over baseline), for a cost of £410 ( + 124% increase over baseline).

Conversely, unlike in the Soton_Suit cases, the impact of the maximum transit time constraint was far smaller. Where the IOW area had fewer sites and was only served by 2 vans in the baseline, there was limited scope to increase the utility of the vehicles and still serve all sites within a single shift period. Hence, allowing more flexibility did not create significant savings, with the best 90 min constrained solution falling within £1 of price of the best 240 min constrained solution (£183).

**Fig. 20:**
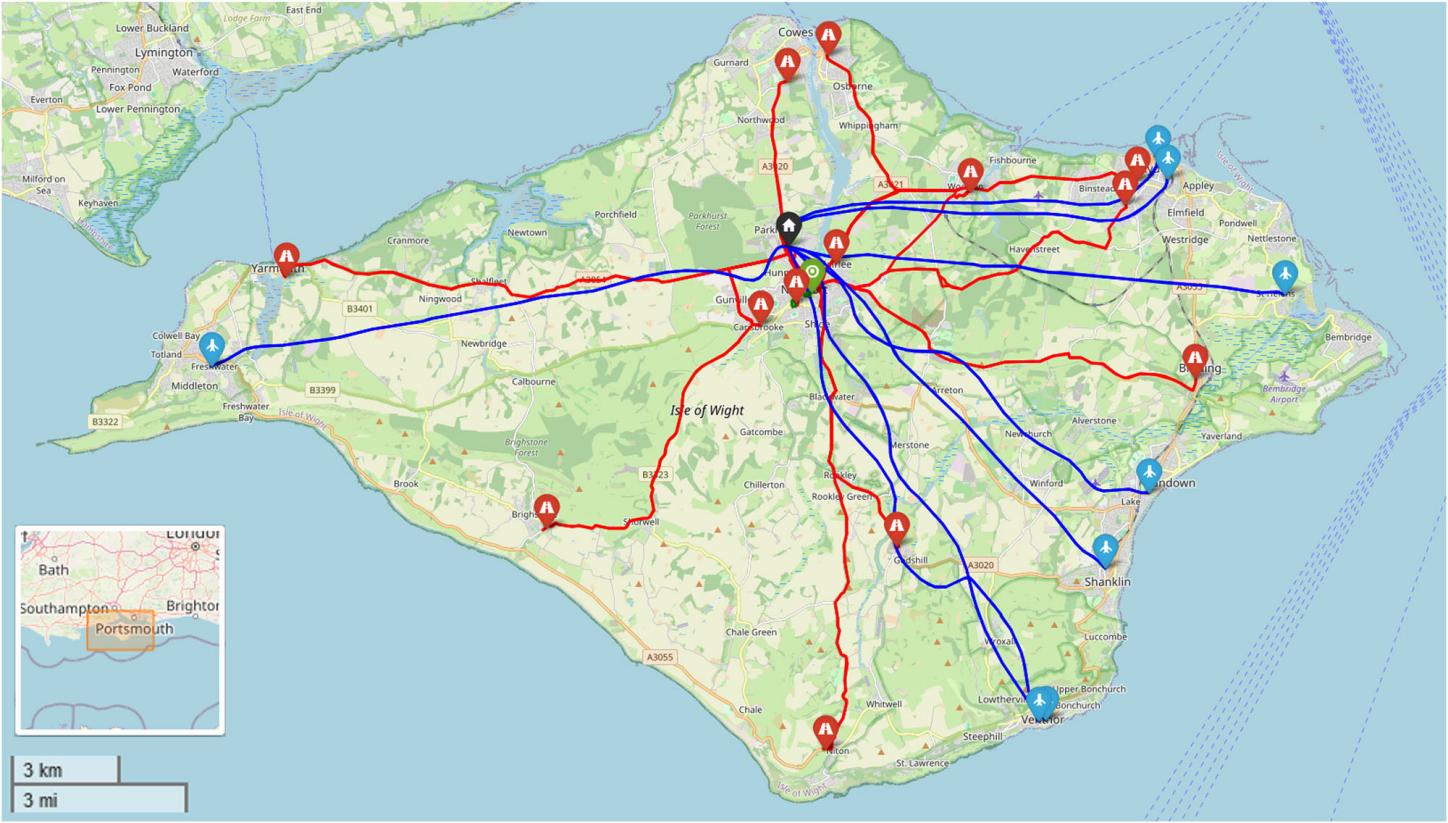
Example routing arrangement at maximum UAV uptake in the IOW_All Scenarios, i.e., UAVs to the furthest sites, vans to the majority of the remainder. Blue, red, and green lines indicate UAV, van routes, and bike routes, respectively. Flight paths may not align with each other due to the methodology used and ground risk varying by time of day. (Base Map © OpenStreetMap contributors).

Aligning with the Soton_All scenarios, when UAVs were permitted to serve all sites in the area, UAVs were selected for the sites with greater van travel times. As before, the maximum time saving was limited by the slowest UAV route (Fig. 20), whilst further uptake was prevented by the inability to remove the remaining van and serve all sites by UAV for the reduced cost.

Within these scenarios, the maximum possible reduction in transit times was 90% (198 min to 19 min). Nonetheless, the 117% (£183 to £410 per shift period, additional £124.7k in operating costs annually) increase in cost should be highlighted as a challenging trade-off, though it may be more appealing if the knock-on benefits support it. Up to two bikes were selected across the full set of solutions, though this was only introduced when the clinic that was limiting the transit time reductions benefitted from cycle consolidation to reduce travel times.

**Fig. 21:**
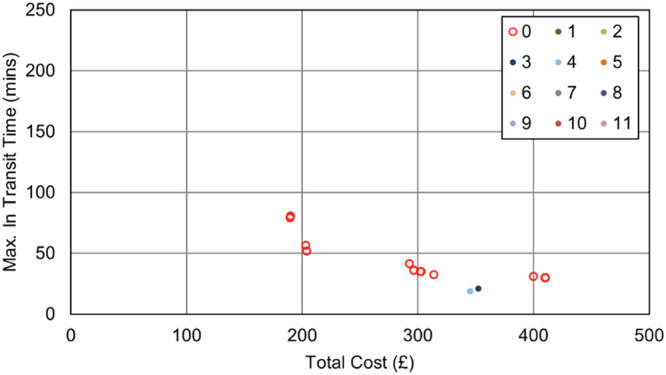
IOW_All_90 – Cost and Maximum Transit Time Trade Relationship. Point colours relate to the number of UAVs in each solution.

**Fig. 22:**
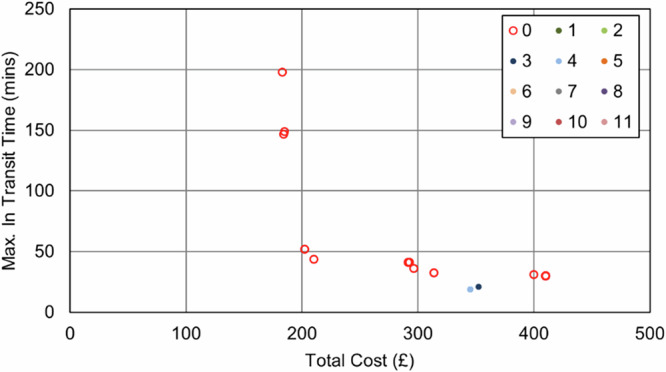
IOW_All_240 – Cost and Maximum Transit Time Trade Relationship. Point colours relate to the number of UAVs in each solution.

The economy of maximising both UAV and operator utility are seen in Fig. 21 and Fig. 22, where no solutions were selected for just 1 or 2 UAVs. Only at a point where 3 UAVs are selected, and a van is removed from the system did introducing UAVs offer sufficient benefits.

### Scottish hebrides

Owing to the challenges around modelling the local arrangements, ferry crossings, and associated timings in this area, only the extremity cases were modelled (i.e., optimised to transit time, or optimised to cost – the baseline case), and a parameter sweep was not adopted in this analysis. Additionally, it was assumed that all sites were suitable for UAV service in all cases. Furthermore, to demonstrate the impact of full UAV uptake, van service was disabled in one scenario.

In the Hebrides, the comparator case was constructed using knowledge of the sites that may require service; however, there were many nuanced, local arrangements that were not possible to capture in a single baseline scenario, including practice staff delivering samples to ferry terminals for onward transit, parcel courier deliveries, and ad-hoc movements by passenger flights to alternative diagnostic labs. Furthermore, the demand from each site and routine collection schedule was not known.

With this in mind, a van, car, and ferry-based comparator case was developed to establish an approximation of a typical business-as-usual collection from all sites. This then enabled a comparison of the intervention scenarios to an equivalent baseline. Nonetheless, it should be highlighted that the comparator is not a true presentation of the business-as-usual.

Unlike in the Southampton and IOW cases, a much longer duration was required for collections due to the greater distances between locations and the longer resulting transit times. As a result, an 8-hour collection shift period was used to ensure the problem was feasible and all samples could be delivered. This assumption follows a similar behaviour in the real-world base case, where it is known that each surgery is served once per day at the most.

**Fig. 23:**
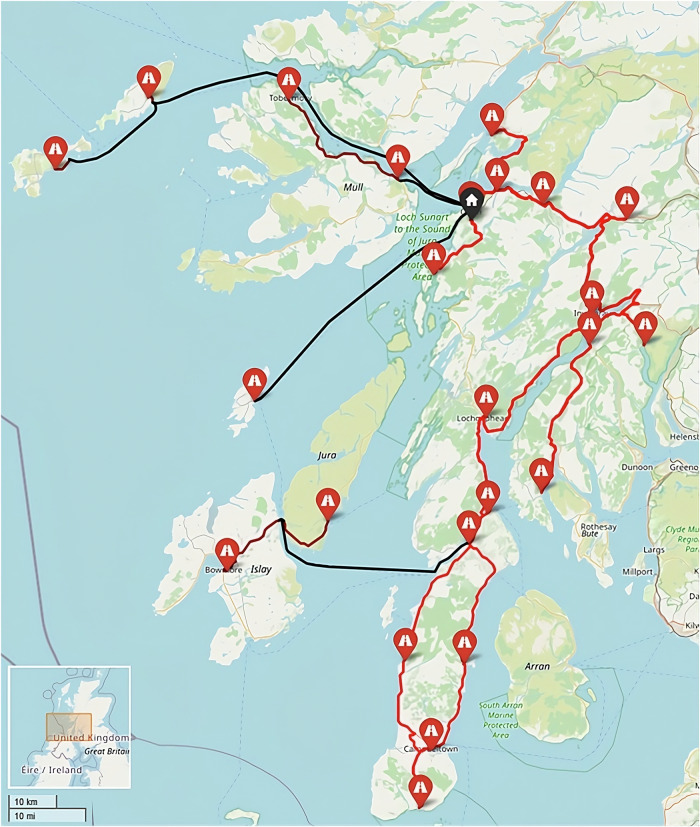
Example routing arrangement in the Hebrides Baseline (Heb_Baseline) Scenario. Red and black lines indicate van/car and ferry routes, respectively. (Base Map © OpenStreetMap contributors).

Under these conditions the Hebrides baseline case (Fig. 23) used 2 vans to visit the 23 locations served in a typical day shift period and complete all collections. Also in the baseline were 6 local arrangements where samples are transported by car to the ferry port by clinic staff, before onward travel in the freight hold of scheduled car ferries. Travelling a collective total distance of 813 miles (1308 km, of which 498 mi (801 km) was van driven), with a total driving time (vans + cars) of 17 h 53 min (including dwell), the cost was estimated to be £476, equating to an annual cost of £119,252 (assuming a single collection each day). The maximum transit time (duration between first pickup in a round and delivery) was 297 min.

**Fig. 24:**
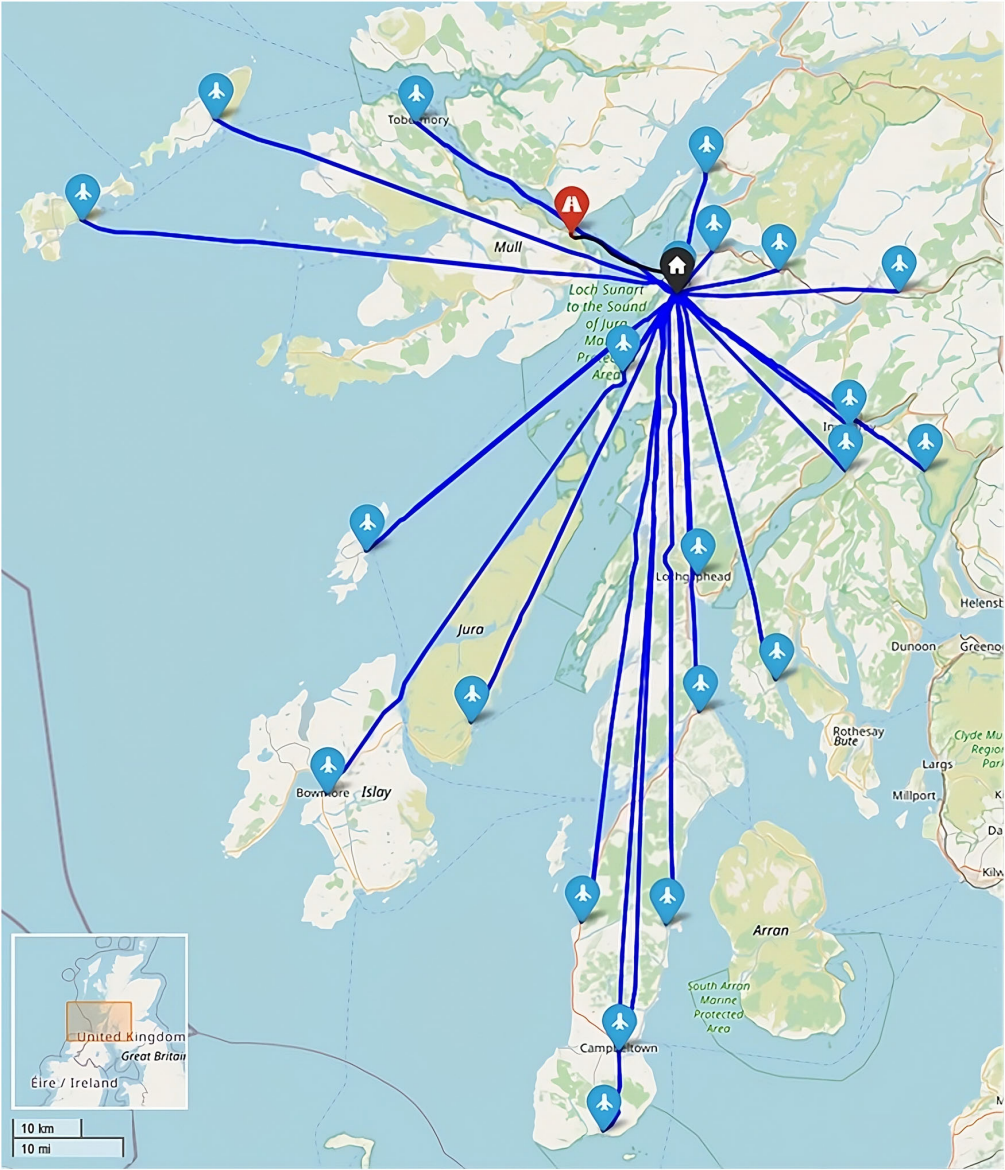
Example routing arrangement in the Heb_All_480 Scenario, i.e., optimised to transit time only. Blue, red, and black lines indicate UAV, car, and ferry routes, respectively. Flight paths may not align with each other due to the methodology used and ground risk varying by time of day. (Base Map © OpenStreetMap contributors).

When considering all sites as suitable for UAV service, it was found that only 1 site (postcode PA65 6BG) remained served by surface transportation when optimising to minimise transit times (Fig. 24). This site remained surface transport served due to being faster to deliver than the furthest UAV-served location in the south (117 min).

The system’s maximum transit time was reduced by 180 min (61%) over the baseline case. Conversely, the cost of the system increased by 132% (£627 per shift period) over the baseline case, with a substantial increase being driven by the introduction of 6 UAVs when only 2 vans were removed. With a UAV operator (who can operate up to 20 UAVs) being 2–3x more costly than each van driver due to the technical capability required and UAVs needing to make single stop trips, replacing only 2 vans with UAVs was unlikely to be cost beneficial.

**Fig. 25:**
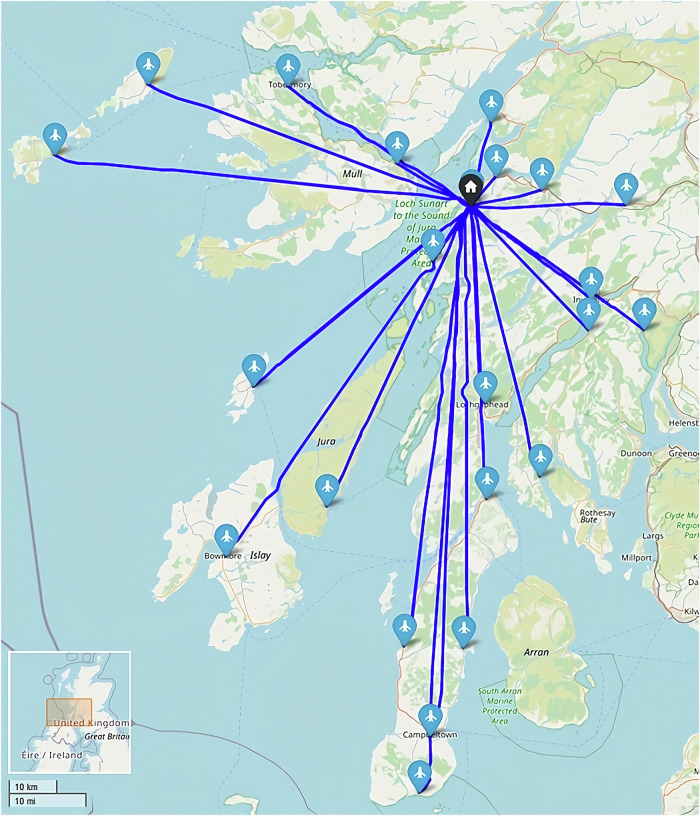
Example routing arrangement at maximum UAV uptake in the Heb_UAVx_480 Scenario, i.e., UAVs to all sites. Blue lines indicate UAV routes. Flight paths may not align with each other due to the methodology used and ground risk varying by time of day. (Base Map © OpenStreetMap contributors).

When all sites were forced to be UAV-served (Fig. 25), transit times did not decrease; however, costs increased by a further 0.9% due to the comparatively expensive additional UAV trip that was introduced. It should be noted that no additional UAVs or operators were required to introduce this change (remained at 6 UAVs, 1 operator).

Whilst not the focus of this paper, it should also be noted that whilst UAV introduction reduces the overall emissions footprint in the area, the emissions impact of serving the island-based sites by UAV is significant when compared to the baseline case. This is due to the dependence of the baseline on ferry services, where the cargo shares a very small portion of the overall footprint, and local transport arrangements that are relatively emissions efficient. This is demonstrated between Heb_UAVx_480 and Heb_All_480, where replacing the only car/ferry-based collection from the Heb_All_480 solution with a UAV service causes an emissions increase from 9.1 kg CO_2_-eq. to 9.4 kg CO_2_-eq. (+3.3%).

### Results summary

Multiple factors were seen to influence the recorded transit times within each of the scenarios, with site suitability being a key characteristic that influenced the potential for the savings offered by UAVs. Across both the Southampton and the IOW case studies, only permitting some sites for UAV uptake caused mainly van-based outcomes to emerge due to the negligible benefit that UAVs could offer in terms of improving delivery time equity.

Transit time limits did not influence the outputs when the objectives were solely weighted towards transit times, but did influence the cost-weighted outcomes, where introducing a 90 min transit time limit caused costs to increase due to additional vehicle assets required in all scenarios.

When optimising to cost instead of transit time, the use of UAVs fell to zero, essentially creating variants of the van-only baseline due to the cost saving available. Furthermore, cycling was only beneficial for reducing maximum transit times where local consolidation could impact the travel times to the sites that were limiting progress towards this objective. To this end, only 3 (Southampton) and 2 (IOW) cycles were ever selected.

Within the larger scale Hebrides case, there was substantially more scope for UAV implementation to improve transit times, owing to the significantly greater distances and times in the baseline case. The dependency on ferry travel typically meant that replacing collection with UAVs would allow times to decrease; however, the need to replace very cost-effective ferry connections with long-distance UAV connections meant that costs were significantly higher than in the baseline.

The upper and lower bounds with respect to cost and transit time within each scenario are given in Table 3, though it should be highlighted that these outputs were extracted from the parameter sweeps, and intermediate solutions offering a compromise of transit time and cost also exist (unless greyed-out).

**Table 3:** Best identified solutions with respect to Transit Times or Cost, by scenario.

	Best ITT Solution (within constraints)						Best Cost Solution (within constraints)					
Scenario ID	Max. ITT (mins)	Cost (£)	Emissions (kg CO_2_-eq.)	Fleet Comp.			Max. ITT (mins)	Cost (£)	Emissions (kg CO_2_-eq.)	Fleet Comp.		
				V*	B	D				V*	B	D
Soton_Baseline							209	377	15.4	4	0	0
Soton_Suit_90	51	824	47.8	8	0	0	85	488	23.2	5	0	0
Soton_Suit_240	51	824	47.8	8	0	0	209	377	15.4	4	0	0
Soton_All_90	33	880	28.9	1	0	10	85	488	23.2	5	0	0
Soton_All_240	33	880	28.9	1	0	10	209	377	15.4	4	0	0
IOW_Baseline							198	183	6.6	2	0	0
IOW_Suit_90	30	410	23.3	4	0	0	90	183	6.3	1	5	0
IOW_Suit_240	30	410	23.3	4	0	0	198	183	6.6	2	0	0
IOW_All_90	19	345	9.4	0	0	4	90	183	6.3	1	5	0
IOW_All_240	19	345	9.4	0	0	4	198	183	6.6	2	0	0
Heb_Baseline							297	476	47.3	2(6)	0	0
Heb_All_480	117	1103	9.1	0(1)	0	6						
Heb_UAVx_480	117	1113	9.4	0	0	6						

^*^Local arrangements in place on islands for short-distance drop-off to ferry terminals. Number in brackets indicates the quantity of these transfers. *ITT* in-transit time, *V* van, *B* bike, *D* UAV/drone.

Results extracted from parameter sweeps, meaning other solutions that offer a compromise of these extremes may also exist.

Furthermore, due to heuristic nature of the solution approach, better solutions *may* exist; however, benchmarking of the logistics planning tool and its underlying model suggests that results are efficient and are near optimal^20,98^.

## Discussion

As noted in Section “Results”, site suitability has substantial impacts on the decision to use UAVs when reducing maximum transit times. As the balance between cost and transit time is shifted towards reducing transit time, the first sites to transfer to UAV service were those at the extremities of the case study areas, typically isolated from other sites, i.e., areas generally with poorer connectivity. When the maximum transit time became limited by a site that could not be consolidated or transferred to UAV service, no further improvements could be made without changing the target metric or landing/service capability.

Section “Methodology and Data” highlighted that maximum transit times were used as the metric to define the level of care standards and ensured that care levels were more equitable. There could be some benefit in using another metric, such as the average transit time, to explore possible improvements to the delivery system speed when only select sites are suitable for UAV delivery; however, this then increases the risk of those sites benefitting from speed improvements whilst the others remain at their existing standard, further increasing inequality within the system.

This study also defined the operational boundary for safe landing around a relaxation of typical aviation regulations and the use of a 5-metre wingspan UAV. When using a winch and cable system (or equivalent) to eliminate the need to land, the scope for improving the equity of delivery remained limited. Another possible solution may be to decrease the size of the UAV or relax the regulations further, although this is likely to present challenges in terms of practicality and safety^12^. Furthermore, if deliveries are required to be fragmented further into smaller shipments to meet the capacity constraints of the platform, any realised transit time saving benefit is substantially reduced^99^.

Industry practitioners have also started to question the likely benefits of implementing UAVs in some environments, such as highly dense urban settings, where alternative modes may offer a simpler and more effective solution than UAVs^58^. In this study, many of these sites were already identified as unsuitable for service due to landing space limitations, or they may be subject to circuitous (less direct) UAV flight path routing due to ground risk challenges; thus, other transport methods may prove more effective for improving transport operations. Such approaches may include full mode shift to cycling^100^, potentially for an increased cost; or consolidation of logistics fleets^101^, potentially at the detriment of transit times.

The transit time constraints in this study represent a theoretical policy position set by healthcare operators to define the minimum standards across all sites. As noted in the Literature Review and Methodology, there are varying limits imposed in different areas and healthcare systems, often based on geographies and budgets.

The findings in Section “Results” indicate that when cost is a consideration, relaxing transit time limits to longer durations (i.e., from 90 min to 240 min) expands the solution space to allow greater flexibility and utilisation of assets, thereby increasing the number of stops per route and reducing empty running at reduced cost. Typical UK NHS standards often define a 4 hour transit time limit^69^, i.e., 240 min; hence, a 90 min limit offers an incremental benefit in terms of delivery speeds. To this end, it was seen that transit time improvements could be made using the existing van-based mode, albeit with additional vehicles to meet the required time limits, and that UAVs were not required in these interventions. As seen in Fig. 15 and Fig. 16, and Fig. 21 and Fig. 22, the longer transit time systems did not feature any UAVs, and only when searching for the shortest transit time systems were any UAVs selected for use – notably at great expense.

With this in mind, it should be highlighted that the time saving benefits cited in many trials may actually be possible through the use of constraints and other modes, before optimisation towards another objective, such as cost or emissions. This may also be considered in combination with an average transit time objective to encourage further uptake of UAVs without creating significant transport inequalities.

Optimising towards transit time only (i.e., cost not considered) resulted in solutions that were not influenced by the transit time limits, as these represented only an upper bound, as would be expected.

As transit times are reduced, the common assumption has been that they will result in an improvement in care quality; however, the true value of this saving in terms of patient care is not well understood.

Whilst the relative benefit of introducing UAVs to the Hebrides is much smaller than in the Southampton and IOW cases (61% vs. 84% and 90%, respectively), the absolute saving may have far greater significance, with delivery times being reduced by up to 3 h. Real-world trials have also identified similar absolute savings, with remote areas in Scotland reportedly benefitting from substantially greater absolute savings of a few hours (2–6 h, ferry dependent, to a reported 15 min)^102,103^, whilst an urban-rural case in England reported a statistically insignificant time saving of 7 min (68 min by ground transport vs. 61 min by UAV)^104^. This can offer potential improvements in patient care equity, with faster delivery from remote locations if an on-demand model is introduced; or the prospect of shorter (compressed) delivery operating shifts. However, these cannot be realised simultaneously due to shorter shifts relying on collections being consolidated into a shorter period of time. The unknown factor in this case is whether the laboratory diagnosis infrastructure has sufficient capacity to process either of these scenarios, and what the long-term impact is for the population in terms of healthcare outcomes.

Time savings may offer advantages if there are quality issues occurring under the business-as-usual case, but unless a more on-demand service is created (i.e., logistics services are provided ad-hoc when specifically required and are not pre-planned), there may be minimal benefit in pursuing these savings. In the Hebrides case study, this shift is most evident, where the current system allows for a maximum of one collection per day, with a strong dependency on local arrangements and unreliable, timetabled ferries^105^. The Hebrides_480 scenarios both give a notable increment in absolute delivery time savings, and the introduction of UAVs gives an additional benefit of dispatch time flexibility (i.e., samples may be sent without timetable restrictions). Nonetheless, implementing an on-demand style delivery service is likely to dramatically increase costs, as was seen in the +132% cost when UAVs were introduced. In the event of UAV operational costs decreasing further, as explored in price sensitivity tests^10^, the relative inefficiency of UAVs with regards to volumes carried per trip makes them far less competitive than other modes and solutions, unless transit time is of utmost importance. Furthermore, adopting an on-demand model will require an increase in the frequency of visits and may mean more UAVs are required and a higher number of flight-hours are incurred. Particularly in higher-demand and higher-population areas, such as Southampton, consumption patterns may change such that the original efficiency gains offered by the technology may be offset by an increased demand in-line with the Jevons Paradox theory^65^. Hence, whilst there may be merit in using a multi-mode system with UAVs in terms of delivery times, any cost benefits will be dependent on removing vans and limiting the service frequency.

Zipline suggests a threshold of 200 deliveries/day for cost saving^106^, involving a need to move to a “focus on ramping up its instant deliveries” to make UAV delivery profitable. Whilst this may be advantageous from a care perspective (i.e., more frequent deliveries), this then changes the baseline comparison to focus on a hypothetical position where the system is operating on an on-demand basis, which may not be appropriate or feasible, particularly where scope for additional spend is limited. In the case study areas in question, it also would not be realistic to serve all sites by UAV due to airspace limitations and likely noise concerns that would arise from the increased air traffic^107–109^. Additionally, within the case studies in this paper, achieving 200 deliveries per day will require substantial re-structuring and consolidation of local healthcare diagnostics networks that would likely not be practical to implement. The restructured networks would possibly necessitate longer travel distances as well, negating the time saving impacts originally sought.

Care must also be taken to consider short term subsidising of services for market growth without long-term sustainability. Many UAV trials have been funded by external parties and have not been proven to be financially sustainable^41,61^, despite reported benefits with regard to matters such as wastage reduction and availability of products. To this end, it is unclear whether it is specifically the UAV service offering the benefit, or just a logistics intervention in general^40^.

This was clearly highlighted by Burchardt and Umlauf^110^ who investigated the hypothesised bottlenecks within the supply chains where Zipline was operating in Africa and found that the rhetoric around the use cases had been significantly misleading. There were notable supply issues at the central distribution sites for emergency products, and routine deliveries of non-urgent medicines were being unnecessarily fulfilled by UAV. The former highlights wider healthcare and supply chain issues, whilst the latter may be a by-product of aiming to realise Zipline’s threshold for delivery viability^106^. Hence, the use of UAVs in this context could be seen as an expedient and inadequate solution that is dwarfed by much larger challenges. Perhaps the most notable in the healthcare environment is an overdependence on just-in-time logistics, which can cause issues with respect to stock-outs and inventory management^110,111^.

Another consideration in the context of transit times is the reliability of operations, and several studies have sought to understand the impact of weather conditions on UAV flyability^102,112^. When aiming to expedite deliveries, establishing a system that cannot operate in poor weather will limit how reliable the delivery times can be. This has been highlighted anecdotally by industry practitioners during a recent UAV trial, where poor weather prevented operations and the hypothesised benefits of faster deliveries^113^. Under the case studies in this paper, this would mean that deliveries could be completed in as little as 33 min (Southampton), 19 min (IOW), or 117 min (Hebrides) when conditions allow, but times will revert to the baseline values (209 min, 198 min, and 297 min) when conditions are too poor to fly. To this end, alternative solutions that compromise on cost and transit time may offer a more reliable operation. For example, 8 vans with shorter routes in Southampton offered a 76% reduction in the maximum transit time (209 min reduced to 51 min), with better weather tolerances than UAVs, and a smaller cost increase of +119%, (+133% when UAVs were used). In the wider healthcare context, many on-demand systems operate with expected delivery timescales (e.g., NHS emergency blood deliveries^114^); thus, reducing delivery timescale reliability will not be advantageous to care outcomes and system management.

In areas where weather conditions are notably more inclement, such as the Hebrides^102^, it has also been suggested that using UAV platforms with higher wind tolerances would improve flyability^112^; hence, also improving operational reliability. This may come at further operational cost but would reliably improve transit times, as demonstrated in this study. Seasonal variations may also present challenges in terms of weather conditions, though it may be that UAVs could provide a supplementary service during periods where weather conditions are better and issues such as tourism may impact local road networks.

The wider societal context must be considered in the introduction of a new transport system. In the case that UAVs were used to serve all sites in the highest demand case study in this paper, Southampton, a UAV would arrive and depart from the hospital on average every 3 min. The additional noise impacts from the 40 low-flying aircraft movements an hour on the community adjacent to the hospital and surrounding area, in addition to the air ambulance service that already visits the hospital, would be substantial^108,109,115^. In the development of trials both in the UK and Australia, there has been a notable resistance and protest against such noise and wildlife impacts^43,116,117^. Those sites that currently experience the greatest transit times are those that would likely benefit most from changing to UAV service in terms of improved care equity and access to transport; however, those in the high demand destinations and along flight routes will likely suffer from increased disruption without necessarily benefiting directly themselves, highlighting another likely trade-off. This also follows a study by Ameso^118^, who discovered that, despite the promise of healthcare improvements, UAV technology is actually causing further challenges through the exacerbation of the underlying labour and infrastructure issues, whilst also not necessarily benefiting all communities affected by their operations.

Another consideration with respect to equitable healthcare and transport is the relative cost of implementation and the size of the population that may benefit. As demonstrated in Section “Results”, the cost of introducing UAVs in all cases is substantial, though when considering the relative population sizes (Southampton c.250,000, IOW c.25,000 people, Hebrides c.8500 (main town)), the rural cases are significantly more costly per capita; however, the relative time and connectivity benefits are also significantly greater, particularly in the Hebrides case. This trade-off is particularly notable as it will likely depend on political influence^119^.

Industry practitioners have also highlighted issues around the scope for transferring UAV operational practices between locations^58^, noting that healthcare often features highly bespoke challenges based on local geographies and demographics, contracting, and funding. Between the case studies in this investigation, there were notable disparities that would likely result in very different transport and healthcare outcomes. For example, the small-scale nature of the IOW network (22 clinics) is likely to limit the economy of scale offered within the Southampton network (76 clinics), making it more challenging to fund; however, the more rural nature of the IOW case potentially offers a greater opportunity for safe UAV implementation. A similar trade-off is seen in Scotland, where the significant distances are likely to benefit from a UAV-based system if a more flexible collection schedule is required (less ferry dependence), though there are notable costs associated with implementing this, and susceptibility to weather influences becomes a far greater challenge.

Beyond healthcare, the likely knock-on impacts of greater UAV delivery adoption may include employment challenges as a result of the increased levels of automation^120^, and the possible proliferation of demand for instant delivery and its associated societal challenges^109,121,122^. Whilst adoption within healthcare use cases may not directly cause these issues, without appropriate management and regulation, it is understood that they are related^38^.

Whilst this study addressed many areas that had previously been neglected in other UAV cost and time analysis studies, some limitations remain. The case study-based methodology used in the investigation means that the results are specific to those exact areas; however, multiple study areas with different characteristics were used to represent a range of different demographical and geographical scenarios. As a result, the findings are not necessarily generalisable for all similar environments and undertaking further research is advisable to establish generalisable relationships between network characteristics, including the clustering and centrality of sites, and the size and shape of the networks; and the recommended combinations of vehicles, including the uptake of UAVs. This could enable a “rule-of-thumb” or lookup to be developed, based on specific objectives, for planners to use when considering changes to their current operations.

This investigation also only modelled one type of UAV, with a given set of characteristics. This UAV was selected based on the case studies’ typical requirements, though some platforms may be capable of faster flight speeds than those which have been modelled. Whilst this would potentially enable further reductions in transit times, the underlying discussion points relating to the value of these improvements and their dependency on the onward supply chain remain. Similarly, other UAV platforms may have different risk profiles that enable more direct flight paths to be considered. Inclusion of risk from all modes (i.e., vans, bikes and UAVs) in the objective function may also provide additional insight regarding the socio-economic cost of transit due to safety, as outlined by Oakey et al.^82^.

It should also be highlighted that UAV staff are assumed to not be shared between case study areas or other delivery operations, possibly reducing their utilisation rates. This was a conservative assumption that helps to account for potentially sporadic demand and task allocation, and helps to address other possible overheads (e.g., warehousing and maintenance time), approvals (e.g., regulator certifications), and training (e.g., dangerous goods), that were not otherwise accounted for in the costings.

## Conclusions

This paper explored the application of UAVs in a multi-mode logistics system (electric vans, UAVs, pedal cycles) for the collection and delivery of medical samples in multiple UK-based case studies (urban/peri-urban/rural/remote). Addressing a knowledge gap relating to understanding the deployment of UAVs in real-world settings, computational modelling was undertaken and suggested that transit time reductions could be made possible through changes to logistics operations across all three case studies; however, these savings did not necessitate the introduction of UAVs and could often be achieved through using additional vans and/or introducing cyclists to consolidate between sites.

The literature review suggested an absence of knowledge relating to UAV use with differing site suitability. Investigating this issue identified that when UAVs were selected for use, locality and site constraints had notable impacts on the take-up rates and subsequently limited the scope for improving the maximum transit time across the network. Where UAVs could only serve those sites that were deemed suitable for UAV service (i.e., sufficient landing space), only vans were selected, transit times could be reduced by up to 84%, though costs increased by 124%. Expanding access for UAVs to serve all sites enhanced this reduction to 90% from the baseline position, though costs also increased (+133% from the baseline).

When considering different restrictions on the maximum transit time, relaxing time limits from 90 min to 240 min enabled slower and more cost-effective solutions to be found, as a result of higher asset utilisation. However, this was only seen where the network was larger in size (urban/peri-urban Southampton), and in smaller cases (peri-urban/rural IOW), utilisation could not be increased further. A longer transit time (8 h) was used in the Hebrides case study (remote and rural), which meant that the baseline case was less costly, though due to the distances involved, transit times could only be reduced by up to 61% by introducing UAVs, with enhanced flexibility.

The sites that typically benefitted most from UAV introductions were those that were geographically furthest from the delivery points, which tended to be the most remote and rural locations in the case studies, due to the longer travel times that occurred in the baseline cases. Despite the potential relative time savings, the absolute savings may offer limited benefits when compared to the associated increases in costs and social challenges, as has been noted in analyses of some of the more developed UAV services across the world. Furthermore, when additional complications such as weather-based flight limitations and bottlenecks in onward care and the supply chain are considered, better alternative solutions to enhance transport systems are likely to exist in many case studies. Thus, accurately conveying the benefits and challenges of using UAV technology is critical to prevent misleading the public into supporting technology that may not be beneficial to all parties, particularly in public sector applications, where the benefits have often been over-stated and longer-term precedents may be set.

## Data Availability

The data sets generated during and/or analysed during the current study are not publicly available for privacy reasons, but are available from the corresponding author on reasonable request.

## Code availability

The underlying code for this study is not publicly available for proprietary reasons, with the exception of the ground risk mapping, which is available as detailed in Pilko et al. (2022). SEEDPOD Ground Risk: A Python application and library for Uncrewed Aerial Systems ground risk analysis and risk-aware path finding. Journal of Open Source Software, 7(71), 4079, 10.21105/joss.04079.
